# ChIP-Seq Analysis of *SlAREB1* Downstream Regulatory Network during Tomato Ripening

**DOI:** 10.3390/foods12122357

**Published:** 2023-06-13

**Authors:** Yanan He, Qiong Wu, Chunxiao Cui, Qisheng Tian, Dongdong Zhang, Yurong Zhang

**Affiliations:** Engineering Center of Ministry of Education, School of Food and Strategic Reserves, Henan University of Technology, Zhengzhou 450001, China

**Keywords:** tomato, abscisic acid, *SlAREB1* transcription factor, ChIP-Seq, fruit ripening

## Abstract

*SlAREB1*, a member of the abscisic acid (ABA) response element-binding factors (AREB/ABFs) family, was reported to play a crucial role in the expression of ABA-regulated downstream genes and affect the ripening of tomato fruit. However, the downstream genes of *SlAREB1* are still unclear. Chromatin immunoprecipitation (ChIP) is a powerful tool and a standard method for studying the interactions between DNA and proteins at the genome-wide level. In the present study, *SlAREB1* was proved to continually increase until the mature green stage and then decrease during the ripening period, and a total of 972 gene peaks were identified downstream of *SlAREB1* by ChIP-seq analysis, mainly located in the intergenic and promoter regions. Further gene ontology (GO) annotation analysis revealed that the target sequence of *SlAREB1* was the most involved in biological function. Kyoto Encylopaedia of Genes and Genomes (KEGG) pathway analysis showed that the identified genes were mainly involved in the oxidative phosphorylation and photosynthesis pathways, and some of them were associated with tomato phytohormone synthesis, the cell wall, pigment, and the antioxidant characteristic of the fruit as well. Based on these results, an initial model of *SlAREB1* regulation on tomato fruit ripening was constructed, which provided a theoretical basis for further exploring the effects of the regulation mechanism of *SlAREB1* and ABA on tomato fruit ripening.

## 1. Introduction

Fruit ripening has attracted much attention because of its particularity to plant bi-ology and its critical impact on fruit quality and shelf life, which involves changes in various appearance and flavor qualities, including color, texture, taste, and aroma [1,2]. Based on the presence or absence of respiration and ethylene transition peaks during ripening, fruit was divided into climactic and non-climactic fruit [3,4]. Tomato, a typical climacteric fruit, was widely used as a model plant for the study of fruit ripening due to its economic importance, obvious ripening period, short life cycle, extensive genome information, and significant metabolic changes [1,5,6,7,8].

Abscisic acid (ABA) is a crucial phytohormone involved in the ripening process of tomato fruit. A number of studies demonstrated that ABA has a positive impact on tomato fruit ripening [1,9,10]. Exogenous ABA treatment was shown to accelerate fruit color transition and firmness reduction and increase ethylene production during tomato ripening, as well as affect the metabolism of sugar and organic acids [11], accumulation of volatile compounds [12], and antioxidant properties [13].

The current ABA signaling model in plant can be described as follows: in the absence of ABA, PP2C inhibits *SnRK2s* activity through physical interactions and phosphatase activity, resulting in the inability of ABA to complete signaling. In the presence of ABA, the binding of ABA molecules to the ABA receptor PYR/PYL/RCAR induces the structural change in the receptor, which allows the ABA receptor to interact with PP2C and inhibit PP2C activity; this leads to the release of *SnRK2s* and, subsequently, activates the downstream ABFs/AREB/ABI5-type bZIP (basic region leucine zipper) transcription factor [14,15]. ABA response element-binding factors (AREB/ABFs), a subfamily of the basic leucine zipper (bZIP) family, are important downstream target genes of *SnRK2*. They can be activated by protein kinases such as *SnRK2.2*, *SnRK2.3*, and *SnRK2.6* and then specifically bind to ABA response elements (ABREs and PyACGTGG/TC) in the promoter regions of downstream target genes to realize the biological regulatory function of ABA in plants [16]. In tomato, two AREB transcription factors, *SlAREB1* and *SlAREB2*, were isolated and identified. Both of them were induced by exogenous ABA treatment, while *SlAREB1* was reported as mainly involved in regulating the expression of stress-related genes and the ripening process in tomato fruit [17,18,19]. Xu et al. [20] found that *SlAREB1* had a strong response to ABA and saline–alkali stress. The *SlAREB1*-mediated ABA signaling pathway may regulate fruit-ripening-related metabolic processes by inducing the expression of genes that encode organic acids (citric acid and malic acid), sugars (glucose and fructose), and amino acid (glutamic acid)-related synthetases in tomato fruit [17,21]. Compared with wild-type tomato fruit, the transcription levels of ethylene synthesis genes *SlACS2*, *SlACS4*, *SlACO1*, and *SlACO3* were significantly increased in *SlAREB1*-overexpresseion fruit and decreased in antisense inhibition lines [21]. Yang et al. [22] found that SNAC9 interacted with *SlAREB1* to affect ABA signaling and further regulate the ripening of tomato fruit and that silencing *SNAC9* fruit would downregulate the expression of *SlACS2* and *SlACO1*. Furthermore, Mou et al. [23] found that SlAREB1 interacted with NOR to promote ethylene synthesis during tomato fruit ripening. These studies suggested that *SlAREB1*-mediated ABA signaling may be involved in the regulation of ethylene biosynthesis and the metabolic processes associated with ripening by inducing the transcription of the corresponding genes and finally affecting tomato fruit ripening. However, the specific regulation mechanism remains to be explicated.

Chromatin immunoprecipitation (ChIP) is a powerful tool and a standard method for studying the interactions between DNA and proteins at the genome-wide level. In recent years, ChIP-Seq technology, which combines ChIP with next-generation high-throughput sequencing technology, has been widely used to identify the target gene regions that transcription factors potentially regulate in plants, owing to its advantages of having a high resolution, a low signal-to-noise ratio, and broad coverage [24,25,26,27,28]. Studies showed that SlAREB1 could regulate tomato fruit ripening, but the downstream target genes that it binds are still unclear. In this study, ChIP-Seq was performed on *SlAREB1*-overexpression, transgenic, mature green tomato fruit to analyze the downstream regulatory network of *SlAREB1*, which could provide references and research ideas for understanding the regulation mechanism of SlAREB1 and ABA on tomato fruit ripening.

## 2. Materials and Methods

### 2.1. Tomato Fruit and Exgenous ABA Treatment

Cherry tomatoes were cultivated in Aisijia Picking Garden in Linying County, Luohe city of Henan Province, China. Approximately 36 days after anthesis, mature green tomato fruit (*Solanum lycopersicum* L.) were manually harvested. Around 600 intact tomato fruit of uniform size were randomly collected at equal height from different plants.

The ABA treatment and storage of tomato fruit were performed per our previous study [12]. Briefly, the collected tomato fruit was sterilized and treated with 1 mM ABA (98%, HPLC, Aladdin) or sterile water (the control) under vacuum (60 kPa) for 180 s and then incubated at 20 °C and 90% relative humidity in darkness for 13 d. During the storage period, tomato fruit was sampled every 3 days, with three batches (8 fruit per batch) randomly sampled per group each time, and the pericarp tissues were frozen with liquid nitrogen and maintained at −80 °C for further use.

Tomato fruit at 6 ripening stages, including immature green (IMG1 and IMG2), mature green (MG), breaker (Br), turning (T), and red ripe (RR), were harvested, and the sampling method was the same as above.

### 2.2. qRT-PCR Analysis

Total RNA was extracted with RNAiso (TaKaRa, Tokyo, Japan), quantified with a BioPhotometer (D30, Eppendorf AG, Hamburg, Germany), and reverse-transcribed to cDNA using the PrimeScript^®^ RT Reagent Kit (DRR047A, TaKaRa, Japan). Primers for selected genes were designed using the Primer 5.0 software, and the obtained sequences are shown in Table S1. The qRT-PCR experiment was performed according to the protocol of TB Green^®^ Premix Ex Taq™ II (RR820, TaKaRa, Japan) using the QuantStudioTM 3 Real-Time PCR Instrument. Actin gene (AK328563.1) was used as the reference gene, and the results were calculated using the 2^−ΔΔCT^ method.

### 2.3. Construction and Identification of SlAREB1-Overexpression Transgenic Tomato Plants

The genomic DNA of the mature green tomato fruit was extracted with high-efficiency plant genomic DNA rapid extraction kit (D200, GeneBetter, Beijing, China), the full length of the open reading frame (ORF) of SlAREB1 was amplified and cloned into pCAMBIA1301 vector via T4 ligase with NcoI and BstEII as double restriction sites, the primers used are shown in S1, and the Flag gene (3 repeats) was inserted in front of the SlAREB1 coding region during the cloning process for subsequence detection and ChIP analysis. The *SlAREB1*-overexpression transgenic tomato plants were obtained via the agrobacterium-mediated method with Micro-Tom plant and Hygromycin B as resistance genes [29], then the positive plants (T0 generation) were cultivated, and PCR analysis was performed both on leaves and mature green fruit of the transgenic plants to verify the successful overexpression of *SlAREB1*. Further ChIP-Seq analysis was carried out on the selected *SlAREB1*-overexpression transgenic mature green fruit.

### 2.4. ChIP-Seq Analysis

ChIP-Seq analysis was conducted following the method of Yang et al. [30] with modifications. Specifically, approximately 4 g of tomato peel from the *SlAREB1*-overexpression transgenic lines were pulverized in liquid nitrogen and cross-linked with 1% formaldehyde at room temperature for 15–30 min, and then 2.5 mL glycine (125 mM) was added to terminate the cross-linking reaction, followed by three washes to remove excess formaldehyde. Afterwards, the chromatin was extracted from the nuclei on ice using a lysis buffer containing protease inhibitors and then sonicated to obtain between 200–500 bp (20 μL of sonicated DNA were used as input sample). For the enrichment of DNA fragments bound to the target protein, 5 μL of Flag antibody (F1804, Sigma, Alexandria, VA, USA) was added to 20 μL of sonicated DNA fragment to form antibody-target protein–DNA complex, and the resulting antibody-target protein–DNA complex was immunoprecipitated using protein G beads (L00277, Sigma, Alexandria, VA, USA). The complex was eluted with elution buffer and subjected to overnight incubation at 65 °C with 20 μL of 5 M NaCl to reverse the cross-linking. Simultaneously, an input sample was mixed with 500 μL of elution buffer and 20 μL of 5M NaCl to decompose the cross-linking and was used as control. Then, the decross-linking product was mingled with 10 μL of 0.5 M EDTA, 5 μL of RNase, 20 μL of Tris-Hcl (pH 7.0), and 2 μL of proteinase K and incubated at 45 °C for 1 h. ChIP DNA Clean and Concentrator™ (Zymo Research Corp., Irvine, CA, USA) was used to purify the ChIP DNA, which was subsequently sequenced using Illumina HiSeq PE150. The quality of obtained reads was assessed with the fastqc software (version: 0.11.5) and filtered using Trimmomatic (version: 0.36). The obtained clean reads were mapped to the tomato genome (https://solgenomics, version:4.0, accessed on 11 May 2023) using BWA software (version: 0.7.15-r1140), and the peak information was analyzed with MACS software (version: 2.1.1.20160309). The entire ChIP-Seq experiment and analysis was performed with the assistance of Aijibaike Biotechnology Co., Ltd., Wuhan, China.

### 2.5. Statistical Analysis

Analysis of variance (ANOVA) and SPSS 22.0 software (IBM, New York, NY, USA) were used to analyze the data of *SlAREB1* gene expression at a significant level of *p* < 0.05. The qRT-PCR results were presented as mean ± standard deviation, and the gene expression plot in this paper was created using Origin 2018 (OriginLab).

## 3. Results

### 3.1. Gene Expression of SlAREB1 during Tomato Fruit Ripening and the Effect of Exogenous ABA Treatment on It

**Figure 1:**
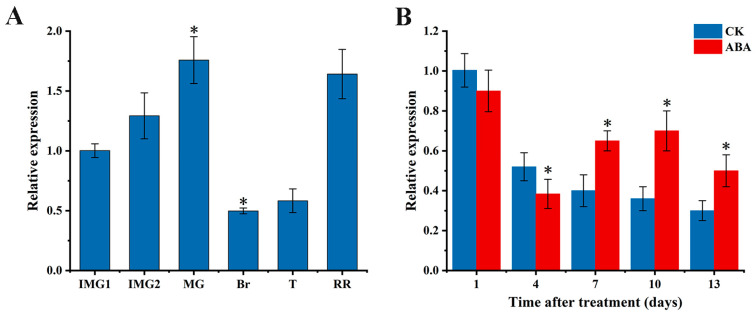
Expression of *SlAREB1* during tomato fruit ripening (**A**) and the effect of exogenous ABA treatment on it (**B**). Error bars represent the standard error (SE) of three biological replicates. In (**A**), the gene expression of *SlAREB1* at the immature green stage is normalized to one, and the asterisk (*) indicates the expression level of the gene in other stage is significantly different from the expression level at IMG1 (*p* < 0.05); in (**B**), the expression of *SlAREB1* in the control group on day 1 is normalized to one, and the asterisk (*) indicates the expression level of the gene in the ABA treatment group is significantly different from that in the control group at the same time (*p* < 0.05).

The transcription levels of *SlAREB1* in tomato fruit at different growth and development stages (immature green, mature green, breaker, turning, and red ripe) are shown in Figure 1A. The expression of *SlAREB1* gradually increased during the early ripening stage, peaked at the mature green stage, declined thereafter, and exhibited a resurgence at the red ripening stage. The effect of exogenous ABA treatment on *SlAREB1* gene expression is shown in Figure 1B, and, compared to the control group, ABA-treated tomato fruit had higher expression levels of *SlAREB1* from the seventh day after ABA treatment. These results suggested that *SlAREB1* may take part in the regulation of tomato fruit ripening, and ABA induced the expression of *SlAREB1* during the ripening process.

### 3.2. Identification of SlAREB1-Overexpression Transgenic Tomato Plants

**Figure 2:**
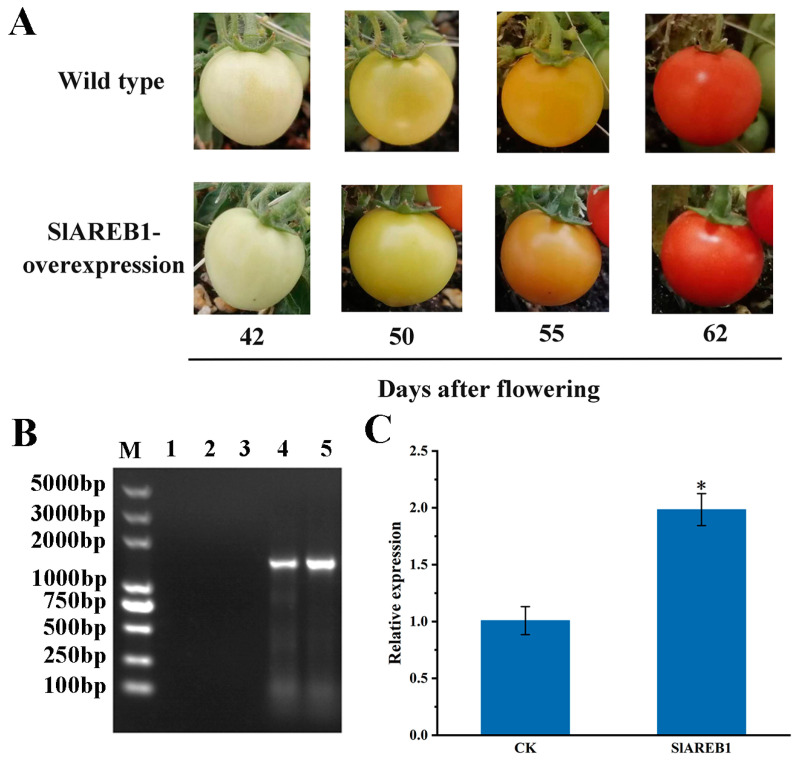
Construction of *SlAREB1*-overexpression transgenic tomato plants. (**A**) Phenotypes between wild-type and *SlAREB1*-overexpression transgenic tomato fruit; (**B**) PCR products’ identification of *SlAREB1*-overexpression transgenic tomato fruit at mature green stage; 1–3 are the control tomato plants, and 4–6 are the transgenic tomato plants; (**C**) qRT-PCR validation of *SlAREB1* overexpression in transgenic tomato fruit. The samples of wild-type tomato fruit at the mature green stage are normalized to one, and the asterisk (*) indicates the *SlAREB1* expression level between the transgenic and wild-type tomato fruit at the mature green stage is significantly different at the significance level of *p* < 0.05.

The results of PCR identification for *SlAREB1*-overexpression transgenic tomato plants are shown in Figure S1. The presence of clear and singular bands in the trans-genic tomato leaves confirmed the successful integration of *SlAREB1* into the genome of transgenic tomato plants. To obtain a clearer view of the effect of *SlAREB1* on tomato fruit ripening, we recorded the phenotypic changes in wild-type and transgenic tomatoes at different times after flowering. As shown in Figure 2A, the growth rates of the transgenic and wild plants were similar, and no obvious difference in phenotype was observed between them. The PCR identification results of *SlAREB1*-overexpression transgenic tomato fruit at the mature green stage are shown in Figure 2B; the clear and single bands in transgenic tomato fruit demonstrating that *SlAREB1* was successfully overexpressed in the transgenic tomato fruit. Moreover, the qRT-PCR results showed that the expression of *SlAREB1* in transgenic tomato fruit was significantly higher than that in wild-type tomato fruit (Figure 2C). Therefore, the transgenic, mature green tomato fruit was suitable for further ChIP-Seq analysis.

### 3.3. ChIP-Seq Analysis

#### 3.3.1. ChIP-Seq Peak Analysis

The overview of the ChIP-Seq data of *SlAREB1* is shown in Table 1, and the raw reads obtained from the two sequenced samples of *SlAREB1*-IP and the input were 52,720,466 and 36,263,534, respectively. The input was the control, which was the genomic DNA after ultrasound interruption. Without immunoprecipitation treatment, the DNA was directly delinked, purified, and analyzed. After quality filtration, 51,618,544 and 35,588,840 clean reads were obtained for *SlAREB1*-IP and the input, respectively. Subsequently, the obtained clean reads were aligned to the tomato genome, and the mapped ratios of *SlAREB1*-IP and the input were 75.23% and 98.14%, respectively.

**Figure 3:**
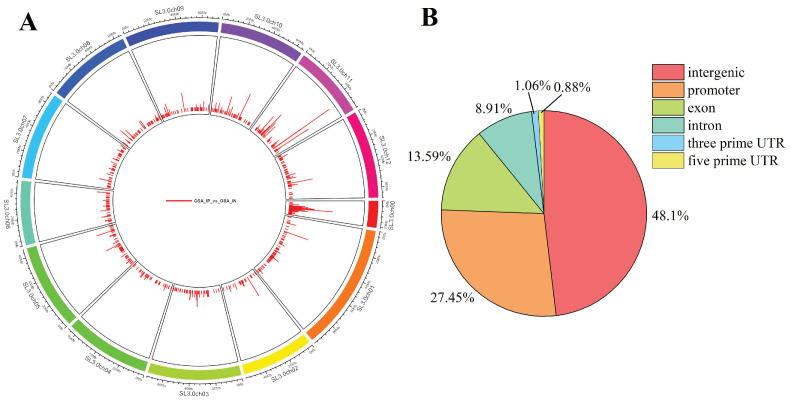
Peak distribution of *SlAREB1* enriched genes. (**A**) Distribution of peaks on the genome. (**B**) Distribution of peaks on gene functional elements.

A total of 972 peaks were enriched and identified in *SlAREB1*-overexpression transgenic tomato fruit (Table S2). These peaks on the genome were distributed in 13 chromosomes (Figure 3A). The distribution of the *SlAREB1* target sequences on the gene functional elements was as follows: 48.1% in the intergenic region, 27.45% in the promoter region, 13.59% in the exon region, 8.91% in the intron region, 1.06% in the 3′-UTR end, and 0.88% in 5′-UTR end (Figure 3B).

#### 3.3.2. Transcription Factor Prediction of Peak-Associated Genes

**Figure 4:**
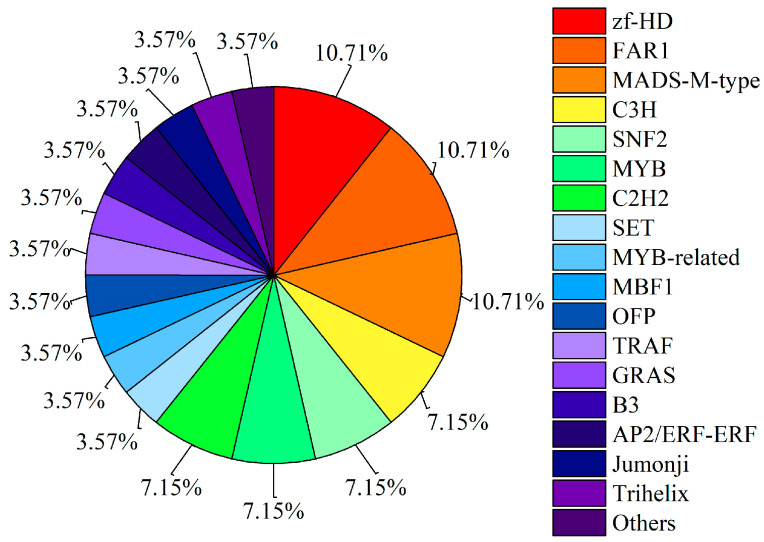
Transcription factor prediction of peak-associated genes.

A total of 28 transcription factors (TFS) were identified in the enriched sequences, which were grouped into 17 TFS families, and the proportion of each TFS family to the total TFS is shown in Figure 4. The identified factors mainly focused on zf-HD-, FAR1-, and MADS-M-type transcription factor families, and the detailed gene information is listed in Table S3.

#### 3.3.3. Gene ontology (GO) and Kyoto Encylopaedia of Genes and Genomes (KEGG) Analysis of SlAREB1 Target Sequences

**Figure 5:**
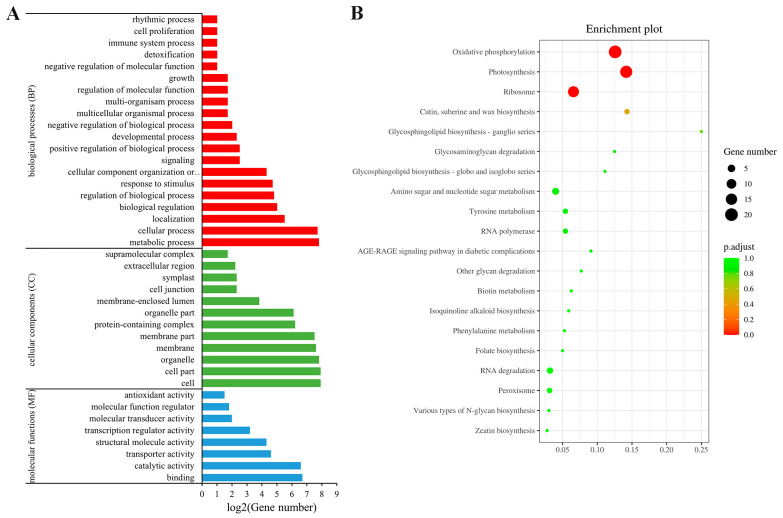
GO (**A**) and KEGG (**B**) analysis of *SlAREB1* target sequences. (**A**) Red represents molecular functions (MF); green represents cellular components (CC); blue represents biological processes (BP).

GO annotation analysis showed that the target sequences of *SlAREB1* were most involved in biological processes and less involved in molecular functions (Figure 5A). In terms of cell components, they were mainly related to the cell, cell part, and organelle. For the biological processes, the enrichment sequences were mainly observed in the metabolic process and cellular process. For the molecular functions, the main functional annotations were involved in binding and catalytic activity.

KEGG pathway analysis provided insights into the metabolic pathways and the specific distribution of the *SlAREB1* target sequences. The top 20 metabolic pathways, as illustrated in Figure 5B, revealed that the *SlAREB1* target sequence was predominantly distributed in the oxidative phosphorylation and photosynthesis pathways.

#### 3.3.4. Downstream Candidate Genes of SlAREB1

For the 972 peak genes obtained above, the relevant information of these genes was analyzed by matching the tomato genome. As shown in Table 2, a total of 20 and 18 genes were found associated with the oxidative phosphorylation and photosynthesis pathways, respectively. Additionally, 8 hormone-related genes (including 3 genes related to ethylene, 1 gene related to auxin, 1 gene related to gibberellin, and 1 gene related to brassinosteroid, respectively), 18 pigment-related genes, 6 cell-wall-related genes, and 3 antioxidant-related genes were identified. More detailed information for all the downstream candidate genes is listed in Table S4.

## 4. Discussion

ABA was proved to be a promoter of tomato fruit ripening. *SlAREB1*, belong-ing to the AREB/ABFs transcription factor family, played an important role in the regulation of ABA downstream gene expression. Studies demonstrated that *SlAREB1* participated in the expression of stress-related genes in tomato fruit and the regulation of fruit ripening [17,18]. In this study, the expression level of *SlAREB1* gradually increased in the early ripening stage of tomato fruit, peaked at the mature green stage, and decreased afterwards. After ABA treatment, the expression level of *SlAREB1* commenced to rise on the seventh day and was significantly higher than that in the CK group, which was consistent with the results of Mou et al. [23], suggesting that *SlAREB1* may play a pivotal role in the regulation of tomato fruit ripening and may actively participate in the ABA-mediated regulatory cascade. In addition, the growth rate and phenotype of *SlAREB1*-overexpression transgenic tomatoes were similar to those of wild-type tomatoes, which was in line with the findings of Bastías et al. [21], indicating that *SlAREB1* is mainly involved in the regulation of metabolic programs during fruit ripening but not for the fruit phenotype.

Fruit ripening and stress were a highly intricate but coordinated process, which were predominantly regulated at the transcriptional level, and transcription factors played a pivotal role in the expression of ripening-related and stress-related genes [31,32]. Hu et al. [33] found that the zf-HD gene family primarily functions in the abiotic stress responses in tomato. In addition, the FAR1 transcription factor family was proved to be implicated in stress responses in tomato fruit, with Solyc09g057880.3 downregulated by ABA treatment and potentially serving a crucial role in stress response [34]. The MADS-box family transcription factors were reported to be involved in diverse developmental processes in plants, particularly in the specification of floral organs, fruit development, and ripening. Among them, Solyc07g017343.1 and Solyc06g034317.1 were found to exhibit distinct expression patterns in different tomato fruit development stages, and they may play a role in fruit development and ripening [35]. In the present study, 28 transcription factors were identified downstream of *SlAREB1*; mainly focusing on zf-HD, FAR1 and MADS-M-type transcription factor families, this indicates that SlAREB1 may be involved in the regulation of metabolic programs and fruit ripening by regulating these transcription factors.

Photosynthesis is a fundamental physiological process in plants that converts light energy into biological energy to maintain growth and development [26]. Mou et al. [36] found that ABA inhibited the expression of most photosynthesis-related genes in the process of tomato fruit ripening, indicating that ABA may modulate fruit ripening through its influence on photosynthesis. Given that *SlAREB1* is an important response factor downstream of the ABA signaling pathway, it is plausible that *SlAREB1* regulates photosynthesis during tomato fruit ripening. In this study, the target sequences of *SlAREB1* were mainly focused on the oxidative phosphorylation and photosynthesis pathways. Notably, studies on postharvest photosynthesis of fruit are limited; therefore, our findings provide a valuable insight into the intricate interplay between photosynthesis and fruit ripening after harvest.

Oxidative phosphorylation is a complex metabolic process that occurs in mitochondria, involving the utilization of energy generated through the oxidation of sugars, lipids, and amino acids to produce adenosine triphosphate (ATP), by facilitating the combination of adenosine diphosphate (ADP) and inorganic phosphate [37], and represents a major pathway for ATP generation in plant cells [37,38]. Wang et al. [39] found that oxidative phosphorylation showed a downward trend during the red-coloring process of strawberry fruit, and the downregulation of the key genes involved in oxidative phosphorylation through Virus-Induced Gene Silencing (VIGS) inhibited respiration and ATP biosynthesis, while promoting the accumulation of sugar, ABA, ethylene, and polyamines (PA), ultimately accelerating strawberry ripening. The oxidative phosphorylation pathway, which includes the electron transport chain and phosphorylation [40], was shown to be linked to the generation of reactive oxygen species (ROS). Additionally, some enzymatic reactions involved in the biosynthesis of antioxidant compounds require energy generated from ATP decomposition [38,41]. Under unfavorable environmental conditions, plants may experience excessive accumulation of ROS, leading to oxidative stress and potential diseases or cytotoxicity from abiotic stress [42,43]. The activity of antioxidant enzymes is crucial in determining a plant’s ability to scavenge ROS. The antioxidant enzymes in plants include superoxide dismutase (SOD), catalase (CAT) [44], peroxidase (POD), polyphenol oxidase (PPO), etc. [13,45]. Glutaredoxin, a small redox protein, was also involved in the ROS scavenging pathways [46]. In the present study, three antioxidant-related genes were identified, namely the glutaredoxin family protein, peroxidase, and laccase. Laccase is a copper-containing polyphenol oxidase that also plays a role in the plant oxidation process [47]. Therefore, *SlAREB1* may potentially affect the antioxidant activity of tomato fruit by interacting with these antioxidant enzymes, which influences the ROS scavenging pathways. These findings suggested that oxidative phosphorylation may play a role during tomato fruit ripening, and *SlAREB1* could potentially impact tomato fruit ripening by modulating the oxidative phosphorylation pathway.

Fruit ripening is accompanied by various changes in taste (sweetness and acidity), texture (softening and firmness), and appearance (color) [48]; fruit color is one of the most important quality attributes of tomatoes that are favored by consumers and is primarily determined by pigments [49,50]. In this study, 18 pigment-related genes were identified to be *SlAREB1* target genes, suggesting that SlAREB1 may potentially interact with these pigment genes to influence tomato fruit color. Furthermore, Wu et al. [51] found that ABA and ethylene synergistically regulated the accumulation of tomato fruit pigments, thereby influencing fruit color development. Since SlAREB1 played a pivotal role in the expression of ABA downstream genes, it is conceivable that it could affect these pigment-related genes, thus contributing to the modulation of tomato fruit color.

Fruit development and ripening are complex processes involved in the inter-play of multiple phytohormones, which can influence fruit quality, nutrition, and taste [52]. Mou et al. [36,53] found that exogenous ABA promoted ethylene biosynthesis and signal transduction by regulating multiple genes in ethylene synthesis and signaling pathways, thereby promoting tomato fruit ripening. 1-Aminocyclopropane-1-carboxylic acid (ACC) synthase [54] and ACC oxidase (ACO) [55] are two key enzymes in ethylene synthesis [56,57]. ACO5, a member of the ACO family, also plays a role in ethylene synthesis [58]. Ethylene response factors (AP2/ERFs) are the response factors in the ethylene signaling pathway, and they can also feedback-regulate the biosynthesis of other plant hormones such as cytokinin, gibberellin, and abscisic acid and are involved in signaling responses to hormones such as auxin, cell division, abscisic acid, and jasmonic acid [59]. In this study, the ACO5 gene and ERF genes were identified as the *SlAREB1* target genes, indicating that *SlAREB1* may influence ethylene biosynthesis and signaling by interacting with the ACO5 gene and ERF genes, thereby regulating tomato fruit ripening. GA-20 oxidase (Gibberellin 20-oxidase) is the key rate-limiting enzyme for gibberellin (GA) biosynthesis, and its synergistic effect is necessary for the growth of tomato fruit [60,61]. Chen et al. [62] found that exogenous gibberellic acid treatment can delay the maturation period of tomato fruit by regulating the transcriptional levels of ethylene-related genes. The auxin response factor (ARF) plays an important role in plant growth and development [63]. Brassinosteroids (BR) is a class of hormones that played an important role in plant growth and development [64]. Zhu et al. [65] found that BR positively regulated tomato fruit ripening and promoted ethylene synthesis. The brassinosteroid hydroxylase may affect the synthesis of brassinosteroid [66]. In this study, a GA-20 oxidase, an auxin response factor-ARF6, and a brassinosteroid hydroxylase were identified among the target genes of *SlAREB1*, but the direct impact of ARF6 on tomato fruit ripening was not demonstrated, indicating that *SlAREB1* may regulate tomato fruit ripening by influencing the biosynthesis of ethylene, gibberellin, and brassinosteroids. Gibberellin and brassinosteroids potentially mediate ethylene synthesis. Further studies are needed to elucidate the exact mechanisms and interactions related to phytohormone biosynthesis and signaling involved in regulating fruit ripening by *SlAREB1* and its target genes.

Fruit firmness is one of the important characteristics of tomato fruit ripening, and the metabolism of the cell wall plays a crucial role in determining the change rate of fruit firmness during ripening [67]. Several enzymes, including endogenous glucanase [68], polygalacturonase (PG) [69,70], expansin [71], pectin methylesterase [72], and PL [73], were shown to be involved in cell wall metabolism and fruit softening [74]. In addition, Zeng et al. [67] found that ABA treatment can accelerate fruit softening and promote the gene expression of β-galactosidase, *SlTBG3*, and *SlTBG4*. In this study, a total of six cell-wall-related genes were identified, including β-galactosidase and pectinesterase., indicating that *SlAREB1* may regulate tomato fruit softening by modulating the expression of cell-wall-related genes, including β-galactosidase and pectinesterase, in response to ABA or other signaling pathways.

**Figure 6:**
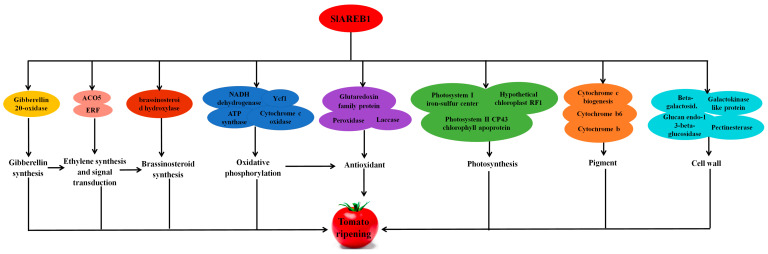
*SlAREB1* regulates tomato fruit ripening model. Different colors represent different anabolic pathways.

Based on the results mentioned above, a regulation model of *SlAREB1* on tomato fruit ripening was drawn (Figure 6). According to the model, *SlAREB1* may primarily regulate genes related to ethylene synthesis and signal transduction, gibberellin synthesis, brassinosteroid synthesis, oxidative phosphorylation, photosynthesis, antioxidants, pigment, and the cell wall, all of which play important roles during tomato fruit ripening. However, the regulatory model of *SlAREB1* on tomato fruit ripening is limited as it was only determined by ChIP-Seq experiments on *SlAREB1*-overexpression transgenic tomatoes. Further experiments are needed to verify its interaction with related genes to clarify the regulatory model of SlAREB1 on tomato fruit ripening. In addition, it is worth noting that many of the target genes of *SlAREB1* in the model have not been extensively studied, so further research is needed to fully validate and refine the proposed model. Additional experimental evidence and functional studies are necessary to elucidate the precise mechanisms by which *SlAREB1* regulates these target genes and their roles in tomato fruit ripening. Further verification and improvement of the regulatory model will contribute to a more comprehensive understanding of the regulatory network governing tomato fruit ripening and the role of *SlAREB1* in this process.

## 5. Conclusions

In this study, the expression level of *SlAREB1* was examined in different stages of tomato fruit ripening, and the influence of ABA on its expression was confirmed, further supporting its role in tomato fruit ripening and its regulation by ABA. Subsequent analysis using ChIP-Seq technology identified the target genes of *SlAREB1*, which were found to be mainly distributed in the oxidative phosphorylation pathway and the photosynthesis pathway. Moreover, *SlAREB1* was found to be involved in the regulation of tomato fruit ethylene synthesis and signal transduction, gibberellin synthesis, brassinosteroid synthesis, the cell wall, pigment, and antioxidant defense. These results provide valuable references and a theoretical basis for further investigations into the mechanism of *SlAREB1* and ABA in tomato fruit ripening.

## Figures and Tables

**Table 1:** Statistical analysis of raw data.

Sample	Raw Reads	Clean Reads	Clean Ratio	Mapped Reads	Map Rate
*SlAREB1*-IP	52,720,466	51,618,544	97.91%	38,834,545	75.23%
Input	36,263,534	35,588,840	98.14%	34,927,951	98.14%

Raw reads: the number of original sequencing reads; clean reads: the number of reads obtained by filtering raw reads; mapped reads: the total number of reads on the alignment; mapped rate: the proportion of the total number of reads on the alignment.

**Table 2:** Target genes of *SlAREB1* (partial).

Gene ID	Subject Length	Subject Start	Subject End	Subject Annotation
**Oxidative phosphorylation**				
Solyc00g013180.1	3901	9,741,922	9,745,822	NADH-ubiquinone oxidoreductase chain 4
Solyc00g014830.3	2241	10,120,367	10,122,607	NADH dehydrogenase subunit 7
Solyc00g019730.2	1112	10,827,171	10,828,282	Cytochrome c oxidase subunit 3
Solyc00g019950.1	1415	10,844,935	10,846,349	NADH dehydrogenase subunit 9
Solyc00g117655.1	195	15,444,467	15,444,661	NADH-ubiquinone oxidoreductase chain 1
Solyc01g020470.2	199	30,837,743	30,837,941	NADH dehydrogenase subunit 9
Solyc01g056670.1	493	55,571,581	55,572,073	NADH dehydrogenase subunit 4L
Solyc03g013460.1	247	45,900,762	45,901,008	Cytochrome c oxidase subunit 3
Solyc03g043610.2	146	7,121,382	7,121,527	ATP synthase subunit a
Solyc05g016220.1	138	15,092,149	15,092,286	Ycf1
Solyc05g023920.1	318	30,101,773	30,102,090	NADH-ubiquinone oxidoreductase chain 1
Solyc07g019510.3	338	11,845,804	11,846,141	Cytochrome c oxidase subunit 1
Solyc08g029260.1	1096	37,265,408	37,266,503	NADH dehydrogenase subunit 2
Solyc10g045750.1	212	35,863,088	35,863,299	NADH-ubiquinone oxidoreductase chain 4
Solyc10g049470.1	157	45,831,441	45,831,597	Ycf1
Solyc11g021240.2	149	13,415,639	13,415,787	Ycf1
Solyc11g021300.1	154	13,419,577	13,419,730	Ycf1
Solyc11g030570.1	339	22,056,502	22,056,840	NADH-ubiquinone oxidoreductase chain 4
Solyc12g035550.1	330	41,911,350	41,911,679	Ycf1
Solyc12g035930.1	153	44,564,598	44,564,750	DNA-directed RNA polymerase subunit beta
**Photosynthesis**				
Solyc00g230070.1	2413	18,659,728	18,662,140	Photosystem II CP43 chlorophyll apoprotein
Solyc01g017090.3	247	23,795,390	23,795,636	NADH-quinone oxidoreductase subunit L
Solyc01g017440.1	143	23,869,851	23,869,993	DNA-directed RNA polymerase subunit alpha
Solyc01g017740.1	146	25,041,926	25,042,071	Cytochrome b6
Solyc01g056870.2	356	57,175,864	57,176,219	Ycf2
Solyc02g011755.1	159	14,135,562	14,135,720	Photosystem I iron-sulfur center
Solyc02g080635.1	291	45,373,848	45,374,138	Photosystem II CP43 reaction center protein
Solyc03g122000.3	437	71,495,017	71,495,453	Cytochrome b6-f complex subunit 4
Solyc04g049003.1	174	38,943,033	38,943,206	Cytochrome c biogenesis protein CcsA
Solyc05g016220.1	138	15,092,149	15,092,286	Ycf1
Solyc10g012230.1	146	4,687,502	4,687,647	Ycf2
Solyc10g047410.1	214	40,732,147	40,732,360	Photosystem II CP43 chlorophyll apoprotein
Solyc10g049470.1	157	45,831,441	45,831,597	Ycf1
Solyc11g018700.2	172	9,107,418	9,107,589	Ycf15
Solyc11g021210.1	152	13,408,194	13,408,345	Cytochrome c biogenesis protein ccsA
Solyc11g021240.2	149	13,415,639	13,415,787	Hypothetical chloroplast RF1
Solyc11g021300.1	154	13,419,577	13,419,730	Hypothetical chloroplast RF1
Solyc12g035550.1	330	41911350	41,911,679	Ycf1
**Phytohormones**				
Solyc07g026650.3	196	30,146,537	30,146,732	ACO5
Solyc09g059510.3	445	54,883,435	54,883,879	ERF
Solyc00g179240.2	171	17,318,514	17,318,684	MADS-box
Solyc10g045690.1	143	35,000,925	35,001,067	Gibberellin 20-oxidase
Solyc12g006350.2	344	870,483	870,826	Auxin response factor 6
Solyc12g006860.2	187	1,281,524	1,281,710	Brassinosteroid hydroxylase
**Pigment**				
Solyc00g019730.2	1112	10,827,171	10,828,282	Cytochrome c oxidase subunit 3
Solyc00g049210.1	378	12,649,895	12,650,272	Cytochrome c-type biogenesis protein CcmF
Solyc01g017740.1	146	25,041,926	25,042,071	Cytochrome b6
Solyc02g021770.1	186	24,252,669	24,252,854	Cytochrome c oxidase subunit 1
Solyc03g013460.1	247	45,900,762	45,901,008	Cytochrome c oxidase subunit 3
Solyc03g013390.1	296	46,389,354	46,389,649	Cytochrome c oxidase subunit 3
Solyc03g122000.3	437	71,495,017	71,495,453	Cytochrome b6-f complex subunit 4
Solyc05g023720.1	248	29,081,746	29,081,993	Apo cytochrome f
Solyc05g025700.1	314	35,921,169	35,921,482	Cytochrome c biogenesis FC
Solyc07g019510.3	274	11,845,109	11,845,382	Cytochrome c oxidase subunit 1
Solyc07g032450.1	167	39,127,895	39,128,061	Cytochrome b6
Solyc09g015880.3	170	11,297,889	11,298,058	Cytochrome c oxidase subunit 2
Solyc09g050020.2	851	35,489,756	35,490,606	Cytochrome b
Solyc11g021210.1	152	13,408,194	13,408,345	Cytochrome c biogenesis protein ccsA
Solyc11g028160.1	432	20,552,580	20,553,011	Cytochrome c biogenesis
Solyc11g039360.1	445	45,467,509	45,467,953	Cytochrome c biogenesis FC
Solyc11g056410.2	277	45,648,871	45,649,147	Cytochrome c oxidase subunit 2
Solyc11g063620.2	262	49,936,635	49,936,896	Cytochrome c biogenesis FN
**Cell wall**				
Solyc07g042220.2	161	55,403,948	55,404,108	Beta-galactosidase
Solyc10g076430.1	152	59,507,919	59,508,070	Pectinesterase
Solyc00g011890.3	171	9,408,568	9,408,738	Galactokinase-like protein
Solyc03g071520.1	191	19,361,038	1,9361,228	Galactosyltransferase family
Solyc05g025500.3	192	32,902,298	32,902,489	Glucan endo-1 3-beta-glucosidase 6
Solyc07g017730.3	202	7,823,023	7,823,224	Glucan endo-1 3-beta-glucosidase 5
**Antioxidant**				
Solyc01g067460.2	207	75,956,536	75,956,742	Glutaredoxin family protein
Solyc02g092580.3	137	54,267,591	54,267,727	Peroxidase
Solyc06g050530.3	191	33,316,524	33,316,714	Laccase

## Data Availability

Sequence data are listed in this article, and some public data are noted in Supplementary Tables S1–S4.

## Supplementary Materials

The following supporting information can be downloaded at https://www.mdpi.com/article/10.3390/foods12122357/s1. Table S1: Primer sequences used in this study. Table S2: Peak information statistics. Table S3: Transcription factor. Table S4: Target genes of SlAREB1. Figure S1. PCR Identification of SlAREB1 transgenic tomato plants. 1–3 were the control tomato plants, 4–5 were the transgenic tomato plants.
