# Supplementary material for: ChIP-Seq Analysis of SlAREB1 Downstream Regulatory Network during Tomato Ripening
Source: Foods. 2023 Jun 13;12(12):2357. doi: 10.3390/foods12122357 (PMC10297250; doi:10.3390/foods12122357)
Supplement: Supplementary file 1 [file foods-12-02357-s001.zip › foods-2381270-supplementary.pdf]

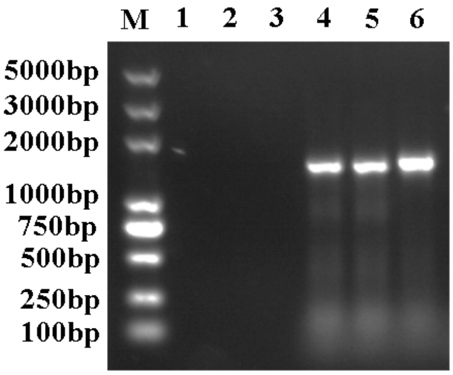

Figure S1. PCR Identification of SlAREB1 transgenic tomato plants. 1-3 were the control tomato plants, 4-5 were the transgenic tomato plants

Table S1. Primer sequences used in this study

**Gene**	**Gene ID**	**F-primer（5'-3'）**	**R-primer（5'-3'）**
RT-SlAREB1	Solyc04g078840	CGGGGAACTTTGGATTGCCG	TGCCCATTAATCCCAGGCCC
Actin	AK328563.1	TGTCCCTATTTACGAGGGTTATGC	CAGTTAAATCACGACCAGCAAGAT
HYG	Flag gene	GGAAGTGCTTGACATTGGGGAG	CGGTGTCGTCCATCACAGTTTG
SlAREB1	Solyc04g078840	GACACAAACTGGTCCATGGATGGTGAGCAAGGGCGAGG	CTGCCGTTCGACGATGGTNACCTTACCATGGACCAGTTTGTGTCC

Table S2. Peak information statistics

**Sample**	**Peak number**	**Total length**	**Average peak length**
SlAREB1-IP	972	373711	384.48

Table S3. Transcription factor

Transcription factor	Number	Gene ID
zf-HD	3	Solyc03g061620.1,Solyc09g057767.1,Solyc05g019985.1
FAR1	3	Solyc01g058003.1,Solyc12g038657.1,Solyc09g057880.3
MADS-M-type	3	Solyc00g179240.2,Solyc07g017343.1,Solyc06g034317.1
C3H	2	Solyc01g014850.3,Solyc12g008890.2
SNF2	2	Solyc01g109970.3,Solyc05g044510.2
MYB	2	Solyc06g009710.3,Solyc05g013430.1
C2H2	2	Solyc04g056320.2,Solyc00g014800.1
SET	1	Solyc06g060380.3
MYB-related	1	Solyc05g013420.1
MBF1	1	Solyc07g062400.3
Others	1	Solyc07g045185.1
OFP	1	Solyc02g085510.1
TRAF	1	Solyc10g079750.2
GRAS	1	Solyc08g078800.2
B3	1	Solyc11g045450.2
AP2/ERF-ERF	1	Solyc09g059510.3
Jumonji	1	Solyc03g097090.3
Trihelix	1	Solyc12g077540.2

Table S4. Target genes of SlAREB1

Gene ID	Subject length	Subject annotation
Solyc00g005000.3	202	Eukaryotic aspartyl protease family protein
Solyc00g005445.1	198	Cyclopropane-fatty-acyl-phospholipid synthase
Solyc00g005907.1	258	50S ribosomal protein L16
Solyc00g006660.1	168	SAUR-like auxin-responsive protein family
Solyc00g006680.2	154	Senescence-associated protein
Solyc00g008040.1	233	alpha/beta-Hydrolases superfamily protein
Solyc00g008570.2	180	SPA1-related 2
Solyc00g008580.1	180	UDP-glucosyl transferase 73B5
Solyc00g009760.2	143	Senescence-associated protein
Solyc00g009765.1	156	Acetolactate synthase
Solyc00g010525.1	156	Tetratricopeptide repeat (TPR)-like superfamily protein
Solyc00g010530.1	173	protein FLOWERING locus D-like protein
Solyc00g011150.1	161	photosystem II stability/assembly factor, chloroplast (HCF136)
Solyc00g011160.2	162	Glucan endo-1,3-beta-glucosidase, basic vacuolar isoform
Solyc00g011673.1	152	ABC transporter G family member 5
Solyc00g011890.3	171	L-arabinokinase
Solyc00g012430.1	151	alpha/beta-Hydrolases superfamily protein
Solyc00g013137.1	534	NADH dehydrogenase subunit 2
Solyc00g013150.1	225	U-box domain-containing protein kinase family protein
Solyc00g013155.1	311	Cytochrome c oxidase subunit 2
Solyc00g013160.2	1290	NADH dehydrogenase subunit 4
Solyc00g013180.1	3901	NADH-ubiquinone oxidoreductase chain 4
Solyc00g014790.2	613	P-loop nucleoside triphosphate hydrolase superfamily protein
Solyc00g014800.1	3149	Zinc finger family protein
Solyc00g014820.2	602	Pentatricopeptide repeat (PPR) superfamily protein
Solyc00g014830.3	2241	NADH dehydrogenase subunit 7
Solyc00g014850.1	7306	Transcription factor jumonji (jmj) family protein / zinc finger (C5HC2 type) family protein
Solyc00g017860.1	144	FAD/NAD(P)-binding oxidoreductase family protein
Solyc00g102000.2	169	Unknown protein
Solyc00g019630.2	1802	F-box/RNI-like superfamily protein
Solyc00g019730.2	1112	Cytochrome c oxidase subunit 3
Solyc00g019740.1	3519	ATPase subunit 8
Solyc00g019950.1	1415	NADH dehydrogenase subunit 9
Solyc00g019970.2	1704	protein kinase family protein
Solyc00g019980.2	373	FAM91 carboxy-terminus protein
Solyc00g020000.1	7500	Mitogen-activated protein kinase
Solyc00g021630.1	6314	NADH-ubiquinone oxidoreductase chain 6
Solyc00g021650.1	3091	Tetratricopeptide repeat (TPR)-like superfamily protein
Solyc00g022070.1	1034	beta glucosidase 8
Solyc00g022090.2	5833	Formyltetrahydrofolate deformylase 1, mitochondrial

Solyc00g022103.1	1242	Transducin family protein / WD-40 repeat family protein
Solyc00g023590.2	8391	Pectin lyase-like superfamily protein
Solyc00g023600.2	460	SERINE CARBOXYPEPTIDASE-LIKE 49
Solyc00g023700.2	3641	Transcription elongation factor SPT5
Solyc00g023710.1	216	Maturase K
Solyc00g025290.1	256	Alpha-1,4 glucan phosphorylase L-2 isozyme
Solyc00g025300.1	247	transmembrane protein
Solyc00g025400.2	420	basic helix-loop-helix (bHLH) DNA-binding superfamily protein
Solyc00g025500.2	278	Pyruvate dehydrogenase, putative
Solyc00g027970.1	211	alpha/beta-Hydrolases superfamily protein
Solyc00g036520.1	164	RNA-binding (RRM/RBD/RNP motifs) family protein
Solyc00g036530.1	2192	P-loop containing nucleoside triphosphate hydrolases superfamily protein
Solyc00g044550.1	617	Ankyrin repeat family protein / BTB/POZ domain-containing protein
Solyc00g044555.1	724	Unknown protein
Solyc00g047190.1	718	Light-independent protochlorophyllide reductase subunit B
Solyc00g049210.1	378	Cytochrome c biogenesis FN
Solyc00g052540.1	3557	RING/FYVE/PHD zinc finger superfamily protein
Solyc00g065510.2	194	S-acyltransferase
Solyc00g068980.2	186	Superoxide dismutase [Cu-Zn] 1
Solyc00g069880.2	3966	SET-domain containing protein lysine methyltransferase family protein
Solyc00g072800.3	316	B-block binding subunit of TFIIIC
Solyc06g050455.1	146	FAR1-related sequence 4
Solyc06g024210.2	152	Senescence-associated protein
Solyc00g090130.2	158	Protein translocase subunit SecA
Solyc00g094520.1	506	NADH-ubiquinone oxidoreductase chain 6
Solyc00g094540.1	3072	Bestrophin-like protein
Solyc00g101700.1	177	Protein kinase
Solyc00g108550.1	243	Homeodomain-like superfamily protein, putative isoform 1
Solyc00g112190.2	2153	Longifolia protein
Solyc00g117655.1	195	NADH-ubiquinone oxidoreductase chain 1
Solyc00g117653.1	999	Glucose-6-phosphate isomerase, cytosolic 1
Solyc00g136565.1	138	2-oxoglutarate-dependent dioxygenase-related family protein
Solyc00g142170.3	3070	Mitovirus RNA-dependent RNA polymerase
Solyc00g153980.1	192	jacalin-related lectin 22
Solyc00g164580.1	2845	phospholipase-like protein (PEARLI 4) family protein
Solyc00g165195.1	663	UDP-glucose pyrophosphorylase 2
Solyc00g179240.2	171	MADS-box transcription factor
Solyc00g181250.2	836	P-loop containing nucleoside triphosphate hydrolases superfamily protein
Solyc00g183050.2	2712	Vacuolar sorting-associated protein 27
Solyc00g203660.2	137	Ribulose bisphosphate carboxylase large chain

Solyc00g210860.1	541	disease resistance protein (TIR class)
Solyc00g218560.1	388	T-box transcription factor, putative (DUF863)
Solyc00g230070.1	2413	Photosystem II CP43 reaction center protein
Solyc00g240580.2	242	P-loop containing nucleoside triphosphate hydrolases superfamily protein
Solyc00g273110.2	146	DNA ligase 4
Solyc00g281110.1	170	inositol polyphosphate kinase 2 alpha
Solyc00g294230.2	195	Disease resistance protein (TIR-NBS-LRR class)
Solyc00g308930.1	148	Lysine-specific histone demethylase 1-like protein
Solyc00g313030.1	370	ARM repeat superfamily protein
Solyc00g320430.1	1048	Vascular endothelial growth factor receptor 2
Solyc01g006730.3	164	Calcium-dependent protein kinase
Solyc01g010797.1	171	F-box family protein
Solyc01g011310.1	203	Eukaryotic aspartyl protease family protein
Solyc01g011393.1	342	U-box domain-containing protein 15
Solyc01g013910.1	193	Endoribonuclease Dicer 2b
Solyc01g014010.1	205	Disease resistance protein (TIR-NBS-LRR class)
Solyc01g014850.3	216	Zinc finger transcription factor 7
Solyc01g014855.1	146	Retrotransposon protein, putative, Ty3-gypsy subclass
Solyc01g016450.1	198	cysteine/histidine-rich C1 domain protein
Solyc01g017090.3	247	NADH dehydrogenase subunit
Solyc01g017200.2	193	Acetyl-coenzyme A carboxylase carboxyl transferase subunit beta, chloroplastic
Solyc01g017220.1	156	ATP synthase epsilon chain, chloroplastic
Solyc01g017333.1	268	NAD(P)H-quinone oxidoreductase chain 4, chloroplastic
Solyc01g017370.1	138	DNA-directed RNA polymerase subunit
Solyc01g017460.2	163	DNA-directed RNA polymerase subunit
Solyc01g017440.1	143	DNA-directed RNA polymerase subunit alpha
Solyc01g017530.1	137	30S ribosomal protein S2, chloroplastic
Solyc01g017740.1	146	Cytochrome b6
Solyc01g019100.1	161	NAD(P)H-quinone oxidoreductase subunit 1, chloroplastic
Solyc01g020430.1	140	Sulfurtransferase
Solyc01g020470.2	199	NADH dehydrogenase subunit 9
Solyc01g022780.1	182	Nascent polypeptide-associated complex subunit beta
Solyc01g033990.1	178	inositol-1,4,5-trisphosphate 5-phosphatase
Solyc01g034023.1	136	Polyketide cyclase/dehydrase and lipid transport superfamily protein
Solyc01g034170.1	216	Urease
Solyc01g028987.1	443	Dead box ATP-dependent RNA helicase, putative
Solyc01g028870.3	143	N utilization substance B
Solyc01g028805.1	190	Cytochrome P450
Solyc01g044270.3	141	DnaJ domain-containing protein
Solyc01g044305.1	371	far-red elongated hypocotyl 1
Solyc01g044373.1	161	HAT family dimerisation domain containing protein
Solyc01g050020.2	139	Photosystem II CP47 reaction center protein

Solyc01g055160.1	359	S-locus F-box protein type-1
Solyc01g056200.1	392	Lactoylglutathione lyase / glyoxalase I family protein
Solyc01g056330.2	151	DNA-directed RNA polymerase subunit beta
Solyc01g056660.2	220	S-locus F-box protein type-9
Solyc01g056670.1	493	NADH dehydrogenase subunit 4L
Solyc01g056720.3	269	small basic intrinsic protein 2.1
Solyc01g056870.2	356	Protein Ycf2
Solyc01g057137.1	418	Retrovirus-related Pol polyprotein from transposon TNT 1-94
Solyc01g057280.2	214	26S proteasome non-ATPase regulatory subunit-like protein
Solyc01g057530.1	199	Pentatricopeptide repeat (PPR) superfamily protein
Solyc01g057570.3	207	RNA-binding (RRM/RBD/RNP motifs) family protein
Solyc01g057585.1	154	Retrovirus-related Pol polyprotein from transposon TNT 1-94
Solyc01g057830.3	140	30S ribosomal protein S1
Solyc01g057960.2	167	Homeobox leucine-zipper protein
Solyc01g058003.1	148	Transposon-like element Lyt2-2 DNA
Solyc01g058220.2	275	F-box family protein
Solyc01g058240.1	137	Ypt/Rab-GAP domain of gyp1p superfamily protein
Solyc01g058327.1	331	Gibberellin 3 beta-hydroxylase family protein
Solyc01g058410.1	326	Cytochrome c oxidase subunit 2
Solyc01g058420.1	216	Ribosomal protein S3
Solyc01g058450.3	153	Flowering time control protein FCA, putative
Solyc01g058500.3	161	TBP-associated factor 2
Solyc01g058675.1	149	Core-2/I-branching beta-1,6-N-acetylglucosaminyltransferase family protein
Solyc01g058703.1	138	Retrovirus-related Pol polyprotein from transposon TNT 1-94
Solyc01g059845.1	150	Retrovirus-related Pol polyprotein from transposon TNT 1-94
Solyc01g059900.3	158	Dirigent protein
Solyc01g060085.1	192	Ribulose bisphosphate carboxylase large chain
Solyc01g060223.1	545	Retrovirus-related Pol polyprotein from transposon 17.6
Solyc01g060425.1	240	Retrotransposon protein, putative, Ty3-gypsy subclass
Solyc01g065915.1	233	Protein CHLOROPLAST ENHANCING STRESS TOLERANCE, chloroplastic
Solyc01g066205.1	150	Retrovirus-related Pol polyprotein from transposon TNT 1-94
Solyc01g066560.3	136	Protein DETOXIFICATION
Solyc01g067460.2	207	Glutaredoxin family protein
Solyc09g064400.2	153	Photosystem I reaction center subunit IX
Solyc01g087490.3	165	Peptidyl-prolyl cis-trans isomerase FKBP42
Solyc01g087970.3	265	Carboxypeptidase
Solyc01g094610.3	173	Rop guanine nucleotide exchange factor, putative
Solyc01g096740.3	195	Protein zinc induced facilitator-like 1
Solyc01g100045.1	308	Methyl-CpG-binding domain-containing family protein
Solyc01g103410.3	173	KH domain-containing protein
Solyc01g105520.3	158	DNA helicase
Solyc01g106943.1	178	Myosin heavy chain-like protein

Solyc01g109970.3	163	SNF2 domain-containing protein / helicase domain-containing protein
Solyc02g043945.1	167	Retrovirus-related Pol polyprotein from transposon TNT 1-94
Solyc02g049070.3	156	4-coumarate:CoA ligase
Solyc02g005350.3	258	succinyl-CoA ligase alpha 2 subunit
Solyc02g005365.1	163	Transposon Ty3-I Gag-Pol polyprotein
Solyc02g005430.2	168	Conglutin beta 7
Solyc02g005510.3	155	Transcriptional coactivator/pterin dehydratase
Solyc02g011755.1	159	Photosystem I iron-sulfur center
Solyc02g011800.1	154	NAD(P)H-quinone oxidoreductase subunit 1, chloroplastic
Solyc02g011990.1	173	Photosystem II protein D1
Solyc02g012000.1	183	ATP synthase subunit alpha, chloroplastic
Solyc02g014700.1	466	histidine kinase 2
Solyc02g014860.3	145	DnaJ domain-containing protein
Solyc02g021390.1	143	ATPase subunit 4
Solyc02g021560.3	194	RNA-binding
Solyc02g021770.1	186	Cytochrome c oxidase subunit 1
Solyc02g023930.1	165	Leucine-rich repeat protein kinase family protein
Solyc02g024000.3	224	WPP domain-associated protein
Solyc02g030580.1	173	RNA polymerase II transcription mediator
Solyc02g031750.3	142	Phloem protein 2-like protein
Solyc02g032173.1	160	Ninja-family protein
Solyc02g032307.1	149	Retrotransposon protein, putative, Ty3-gypsy subclass
Solyc02g063080.1	171	NAD(P)H-quinone oxidoreductase subunit K, chloroplastic
Solyc02g067590.1	209	DNA-directed RNA polymerase subunit beta
Solyc02g077860.1	149	Ribulose bisphosphate carboxylase large chain
Solyc02g080280.2	201	NAD(P)H-quinone oxidoreductase subunit 1, chloroplastic
Solyc02g080330.3	167	Cytochrome P450 family protein
Solyc02g080635.1	291	Photosystem II CP43 reaction center protein
Solyc02g085510.1	161	ovate family protein 1
Solyc02g092580.3	137	Peroxidase
Solyc03g025760.3	145	Protein O-glucosyltransferase 1
Solyc03g026130.3	161	Cytochrome P450
Solyc03g043610.2	146	ATP synthase subunit a
Solyc03g044810.2	157	Methyl jasmonate esterase
Solyc03g071507.1	163	Kinase interacting (KIP1-like) family protein
Solyc03g071520.1	191	Hexosyltransferase
Solyc03g071710.1	153	Cyclin-dependent protein kinase inhibitor Siamese
Solyc03g071720.3	150	Phytol kinase
Solyc03g071790.1	322	Defective in meristem silencing 3
Solyc03g051590.2	146	Protein Ycf2
Solyc03g051623.1	147	Transposon Ty3-I Gag-Pol polyprotein
Solyc03g053100.3	136	Kinesin-like protein
Solyc03g058210.2	344	50S ribosomal protein L14, chloroplastic
Solyc03g058300.1	533	F-box and associated interaction domains-containing protein

Solyc03g058920.3	168	Mitochondrial outer membrane porin
Solyc03g061540.1	718	NADH dehydrogenase subunit 2
Solyc03g061550.1	2554	poly ADP-ribose polymerase 3
Solyc03g061620.1	285	Zinc finger family protein
Solyc03g061640.1	175	Chromatin remodeling protein, putative
Solyc03g061655.1	305	Ribosomal protein S12
Solyc03g063010.1	231	cysteine-rich RECEPTOR-like kinase
Solyc03g063070.1	209	vacuolar protein sorting-associated protein, putative (DUF1162)
Solyc03g063510.1	676	NADH dehydrogenase subunit 2
Solyc03g063515.1	758	HAT family dimerisation domain containing protein, expressed
Solyc03g063550.1	326	cotton fiber protein
Solyc03g063750.3	182	exostosin family protein
Solyc03g064040.1	199	ubiquitin-specific protease 16
Solyc03g065205.1	275	DUF1336 family protein, putative (DUF1336)
Solyc03g065230.1	160	Transposon protein, putative, CACTA, En/Spm sub-class
Solyc03g065250.3	162	Fatty acid hydroxylase superfamily protein
Solyc03g013610.1	164	50S ribosomal protein L32, chloroplastic
Solyc03g013615.1	164	NAD(P)H-quinone oxidoreductase subunit I, chloroplastic
Solyc03g013605.1	385	30S ribosomal protein S18, chloroplastic
Solyc03g013470.1	321	DNA polymerase
Solyc03g013460.1	247	Cytochrome c oxidase subunit 3
Solyc03g013390.1	296	Cytochrome c oxidase subunit 3
Solyc03g013377.1	136	Retrovirus-related Pol polyprotein from transposon TNT 1-94
Solyc03g078005.1	250	Photosystem I P700 chlorophyll a apoprotein A1
Solyc03g078070.2	197	Glycine dehydrogenase (decarboxylating) 1, mitochondrial
Solyc03g078330.1	175	3-ketoacyl-CoA synthase
Solyc03g082685.1	162	Neuroblastoma-amplified protein
Solyc03g083610.3	151	UMP-CMP kinase
Solyc03g083665.1	428	transmembrane protein
Solyc03g095380.2	149	Unknown protein
Solyc03g097090.3	162	Lysine-specific demethylase
Solyc03g097350.3	155	Kinase family protein
Solyc03g097886.1	147	50S ribosomal protein L14, chloroplastic
Solyc03g112520.3	201	FKBP12-interacting of 37 kDa-like protein
Solyc03g115380.2	156	UDP-glucose 6-dehydrogenase family protein
Solyc03g115720.3	142	zinc finger (C3HC4-type RING finger) family protein
Solyc03g116370.3	324	RP/EB family microtubule-associated protein
Solyc03g118525.1	147	ATP-dependent helicase/deoxyribonuclease subunit B
Solyc03g119170.3	179	Ribosomal protein S24/S35, mitochondrial
Solyc03g122000.3	437	Cytochrome b6-f complex subunit 4
Solyc04g005330.3	172	Alpha-1,4-glucan-protein synthase
Solyc04g005830.3	176	P-loop containing nucleoside triphosphate hydrolases superfamily protein

Solyc04g011620.3	293	Peptidyl-prolyl cis-trans isomerase FKBP20-1 (AHRD V3.3 *** FK201_ARATH)
Solyc04g012200.1	142	Protein kinase superfamily protein
Solyc04g016170.2	195	Photosystem I P700 chlorophyll a apoprotein A2
Solyc04g018035.1	283	Ty3-gypsy retrotransposon protein
Solyc04g018140.1	149	F-box/RNI-like superfamily protein
Solyc04g018143.1	148	Retrotransposon protein, putative, Ty3-gypsy subclass
Solyc04g018175.1	166	Plant L-ascorbate oxidase
Solyc04g039850.1	349	ATP synthase subunit beta
Solyc04g026105.1	141	Retrovirus-related Pol polyprotein from transposon TNT 1-94
Solyc04g025993.1	170	Zinc finger, RanBP2-type
Solyc04g025400.3	146	Lipid phosphate phosphatase
Solyc04g025375.1	245	Retrovirus-related Pol polyprotein from transposon TNT 1-94
Solyc04g025288.1	192	chromatin remodeling 38
Solyc04g025120.2	140	ATPase
Solyc04g045470.3	165	O-fucosyltransferase family protein
Solyc04g045475.1	284	O-fucosyltransferase family protein
Solyc04g045640.1	183	Pyrophosphate--fructose 6-phosphate 1-phosphotransferase subunit alpha 1
Solyc04g047880.1	136	RING/U-box superfamily protein
Solyc04g049003.1	174	Cytochrome c biogenesis protein CcsA
Solyc04g049090.3	159	SlMlo1
Solyc04g049240.1	173	Photosystem II protein D1
Solyc04g049250.1	184	Transport membrane protein
Solyc04g049620.2	159	2-oxoglutarate (2OG) and Fe(II)-dependent oxygenase superfamily protein
Solyc04g049673.1	171	50S ribosomal protein L20, chloroplastic
Solyc04g049780.2	148	Retrovirus-related Pol polyprotein from transposon TNT 1-94
Solyc04g050430.1	408	60S ribosomal protein L12
Solyc04g050440.3	175	ammonium transporter
Solyc04g050800.2	305	G-type lectin S-receptor-like serine/threonine-protein kinase At5g24080
Solyc04g050803.1	144	Retrovirus-related Pol polyprotein from transposon TNT 1-94
Solyc04g051270.2	216	CASP-like protein
Solyc04g054513.1	155	Retrovirus-related Pol polyprotein from transposon TNT 1-94
Solyc04g056320.2	218	Protein SENSITIVE TO PROTON RHIZOTOXICITY 1
Solyc04g072265.1	197	Midasin
Solyc04g080730.3	250	mitogen-activated protein kinase 9
Solyc04g082490.3	160	Niemann-Pick C1
Solyc04g082990.3	191	Tetratricopeptide repeat protein 37
Solyc05g005130.2	202	Disease resistance protein, putative
Solyc05g007720.1	319	Helicase/SANT-associated, DNA binding protein
Solyc05g008560.2	193	Pentatricopeptide repeat-containing protein, putative
Solyc05g010400.3	174	NSP-interacting kinase 1

Solyc05g013420.1	242	myb-like transcription factor family protein
Solyc05g013430.1	147	myb-like transcription factor family protein
Solyc05g013510.3	177	Phosphate transporter
Solyc05g013530.3	245	Octicosapeptide/Phox/Bem1p domain-containing protein kinase
Solyc05g014570.1	199	MYB family transcription factor
Solyc05g014880.2	553	Histone H2A
Solyc05g015715.1	270	HAT family dimerisation domain containing protein
Solyc05g015945.1	156	hydroxysteroid dehydrogenase 2
Solyc05g015990.1	161	serine carboxypeptidase-like 43
Solyc05g016120.2	210	Photosystem II protein D1
Solyc05g016220.1	138	Ycf1
Solyc05g016230.3	164	Protein BREAST CANCER SUSCEPTIBILITY 1-like protein
Solyc05g016727.1	175	HAT family dimerisation domain containing protein
Solyc05g018595.1	207	Retrovirus-related Pol polyprotein from transposon TNT 1-94
Solyc05g018860.1	337	DCD (Development and Cell Death) domain protein
Solyc05g018910.2	266	Kinase, putative
Solyc05g019980.2	141	trichome birefringence-like protein (DUF828)
Solyc05g019985.1	154	ZF-HD homeobox protein
Solyc05g020010.1	225	Photosystem I P700 chlorophyll a apoprotein A2
Solyc05g020047.1	194	Transport membrane protein
Solyc05g021247.1	156	LINE-1 reverse transcriptase like
Solyc05g021410.2	159	histone-lysine N-methyltransferase SUVR5
Solyc05g021480.1	162	homeobox-leucine zipper protein ATHB-8
Solyc05g023610.1	141	cadherin EGF LAG seven-pass G-type receptor, putative (DUF3527)
Solyc05g023720.1	248	Cytochrome f
Solyc05g023775.1	159	NADH-quinone oxidoreductase protein
Solyc05g023920.1	318	NADH-ubiquinone oxidoreductase chain 1
Solyc05g024100.1	194	Lysine-specific histone demethylase 1-like protein
Solyc05g024245.1	175	Enoyl CoA hydratase/isomerase
Solyc05g025500.3	192	Glucan endo-1,3-beta-glucosidase, putative
Solyc05g025580.1	342	N-glycosylase/DNA lyase OGG1
Solyc05g025670.1	736	Respiratory burst oxidase, putative
Solyc05g025700.1	314	Cytochrome c biogenesis FC
Solyc05g025820.3	218	Kinase family protein
Solyc05g026200.1	146	Acetyl-coenzyme A carboxylase carboxyl transferase subunit beta, chloroplastic
Solyc05g026510.3	370	Sister-chromatide cohesion protein 3
Solyc05g032750.3	150	EEIG1/EHBP1 protein amino-terminal domain protein
Solyc05g032730.1	186	Protein DETOXIFICATION
Solyc05g039960.1	175	NHL domain-containing protein, putative
Solyc05g041170.2	270	Mediator of RNA polymerase II transcription subunit 20-like protein
Solyc05g041200.3	200	4-hydroxyphenylpyruvate dioxygenase
Solyc05g041230.1	168	Photosystem II CP43 reaction center protein

Solyc05g041400.1	619	NADH:ubiquinone oxidoreductase, subunit 1/F420H2 oxidoreductase subunit H
Solyc05g042110.1	159	Disease resistance protein
Solyc05g044510.2	261	RNA helicase DEAH-box17
Solyc05g044570.2	153	Helicase protein with RING/U-box domain-containing protein
Solyc05g045730.1	306	transcriptional regulator EFH1-like protein
Solyc05g045740.1	941	P-loop containing nucleoside triphosphate hydrolases superfamily protein
Solyc05g045750.2	377	Maturase K
Solyc05g045777.1	559	Cytochrome c oxidase subunit 2
Solyc05g046287.1	159	At2g11520-like protein
Solyc05g047580.3	139	Terpene cyclase/mutase family member
Solyc05g050820.3	202	DNAJ
Solyc05g052640.3	311	Mitochondrial substrate carrier family protein
Solyc05g054710.3	173	Beta-hexosaminidase
Solyc05g055040.3	152	Hexosyltransferase
Solyc06g005600.3	143	Scarecrow-like transcription factor 11, putative isoform 1
Solyc06g009710.3	230	R2R3MYB transcription factor 111
Solyc06g009940.1	184	Photosystem I P700 chlorophyll a apoprotein A1
Solyc06g009980.1	148	Nucleotide-diphospho-sugar transferases superfamily protein
Solyc06g010165.1	372	Nucleotide/sugar transporter family protein
Solyc06g010170.3	191	lysine ketoglutarate reductase trans-splicing protein (DUF707)
Solyc06g010260.3	242	SNF1-related protein kinase regulatory subunit beta-2
Solyc06g010270.1	141	Pentatricopeptide repeat-containing protein, putative
Solyc06g011490.3	181	Unknown protein
Solyc06g024380.1	187	DNA polymerase epsilon catalytic subunit
Solyc06g024375.1	175	Cytochrome c oxidase subunit 1
Solyc06g024310.1	343	Elongation defective 1 family protein
Solyc06g024240.1	167	ribosomal protein 1
Solyc06g024245.1	177	Class II aaRS and biotin synthetases superfamily protein
Solyc06g016700.1	468	2-oxoglutarate (2OG) and Fe(II)-dependent oxygenase superfamily protein
Solyc06g016765.1	171	Reverse transcriptase
Solyc06g018050.3	146	mitochondrial calcium uniporter complex protein-like
Solyc06g030630.1	174	Delta-aminolevulinic acid dehydratase, chloroplastic
Solyc06g031740.2	249	ABC transporter G family member 22
Solyc06g032740.1	191	Protein Ycf2
Solyc06g033782.1	178	Retrovirus-related Pol polyprotein from transposon TNT 1-94
Solyc06g035555.1	147	MADS-box transcription factor
Solyc06g036020.1	158	kinesin-like protein 1
Solyc06g036130.3	178	Protein DETOXIFICATION
Solyc06g036210.1	142	NAD(P)H-quinone oxidoreductase subunit K, chloroplastic
Solyc06g036505.1	143	Retrovirus-related Pol polyprotein from transposon TNT 1-94
Solyc06g036580.3	175	U4/U6 small nuclear ribonucleoprotein Prp3

Solyc06g036770.1	645	CASP-like protein
Solyc06g036803.1	284	Skp1
Solyc06g043038.1	177	Ycf68
Solyc06g042990.1	232	ATPase subunit 4
Solyc06g048480.3	177	Transducin/WD40 repeat protein
Solyc06g048680.2	322	GDSL esterase/lipase
Solyc06g050490.1	178	Disease resistance protein (CC-NBS-LRR class) family protein, putative
Solyc06g050530.3	191	Laccase
Solyc06g050925.1	180	Retrovirus-related Pol polyprotein from transposon TNT 1-94
Solyc06g051330.1	142	IQ-domain 30
Solyc06g053430.3	171	selenoprotein family protein
Solyc06g060380.3	139	Sister chromatid cohesion PDS5-like protein
Solyc06g062375.1	163	Acid phosphatase 1
Solyc06g064450.3	185	Cytoplasmic membrane protein
Solyc06g068840.3	164	Calcium-dependent phospholipid-binding Copine family protein
Solyc06g072380.3	182	3,4-dihydroxy-2-butanone kinase
Solyc06g072540.1	150	ATP synthase subunit alpha, chloroplastic
Solyc06g074040.1	311	Late embryogenesis abundant (LEA) hydroxyproline-rich glycoprotein family
Solyc06g074590.2	241	Protein Ycf2
Solyc06g074600.1	165	DNA-directed RNA polymerase subunit beta
Solyc06g084624.1	139	DWNN domain, a CCHC-type zinc finger
Solyc07g004993.1	184	Phosphatidylinositol N-acetyglucosaminlytransferase subunit P-like protein
Solyc07g007710.3	148	Defensin protein
Solyc07g008170.2	194	Methyl-CpG-binding domain protein
Solyc07g008760.3	210	Tetratricopeptide repeat-containing protein
Solyc07g008990.1	259	Photosystem II D2 protein
Solyc07g009350.3	144	U3 small nucleolar RNA-associated protein
Solyc07g017343.1	142	MADS-box transcription factor
Solyc07g017730.3	202	Glucan endo-1,3-beta-glucosidase, putative
Solyc07g018387.1	158	Photosystem II reaction center protein I
Solyc07g019480.1	150	B3 domain-containing protein family
Solyc07g019500.2	152	B3 DNA-binding domain protein
Solyc07g019505.1	274	Cytochrome c oxidase subunit 1
Solyc07g019510.3	338	Cytochrome c oxidase subunit 1
Solyc07g019520.1	147	Isocitrate lyase
Solyc07g020807.1	271	Retrovirus-related Pol polyprotein from transposon TNT 1-94
Solyc07g021110.1	196	Protein Ycf2
Solyc07g021120.2	152	conserved telomere maintenance component 1
Solyc07g021330.2	322	Retrovirus-related Pol polyprotein from transposon TNT 1-94
Solyc07g021660.2	445	Ubiquitin-conjugating enzyme family protein
Solyc07g024040.2	171	Photosystem I reaction center subunit VIII

Solyc07g025130.1	448	Ribosomal protein L2
Solyc07g025450.2	137	histidine kinase 1
Solyc07g026600.1	293	plant-specific transcription factor YABBY family protein
Solyc07g026630.1	138	Cysteine/Histidine-rich C1 domain family protein
Solyc07g026650.3	196	1-aminocyclopropane-1-carboxylate oxidase 5
Solyc07g026725.1	176	Retrovirus-related Pol polyprotein from transposon TNT 1-94
Solyc07g032020.2	289	DNA-directed RNA polymerase subunit
Solyc07g032075.1	220	50S ribosomal protein L20, chloroplastic
Solyc07g032290.2	155	Tryptophan/tyrosine permease
Solyc07g032450.1	167	Cytochrome b6
Solyc07g037960.1	149	Ectonucleotide pyrophosphatase/phosphodiesterase, putative
Solyc07g039195.1	221	Dystrophin-1
Solyc07g039585.1	150	HAT family dimerisation domain containing protein, expressed
Solyc07g040970.1	317	ribosomal protein L5
Solyc07g041300.2	214	Spermidine synthase
Solyc07g041513.1	174	UDP-Glycosyltransferase superfamily protein
Solyc07g041630.1	146	NADH dehydrogenase subunit 2
Solyc07g041970.3	229	Subtilisin-like protease
Solyc07g042220.2	161	Beta-galactosidase
Solyc07g045185.1	253	CONSTANS-like zinc finger protein
Solyc07g045230.3	272	U-box domain-containing protein
Solyc07g049220.3	153	Serine/threonine protein phosphatase 2A regulatory subunit B
Solyc07g052065.1	217	Retrovirus-related Pol polyprotein from transposon TNT 1-94
Solyc07g052880.3	359	Formin-like protein
Solyc07g061900.3	151	50S ribosomal protein L4, putative
Solyc07g062400.3	139	multiprotein bridging factor 1c
Solyc07g063260.3	182	MLO-like protein
Solyc07g063350.3	170	COP1-interacting protein, putative
Solyc08g006025.1	160	NAC domain-containing protein
Solyc08g006254.1	181	serine/threonine-protein kinase AFC1
Solyc08g006256.1	160	serine/threonine-protein kinase AFC1
Solyc08g015810.2	141	GDA1/CD39 nucleoside phosphatase family protein
Solyc08g015965.1	189	Retrovirus-related Pol polyprotein from transposon TNT 1-94
Solyc08g016080.3	322	high chlorophyll fluorescence phenotype 173
Solyc08g036550.1	239	disease resistance protein (TIR class)
Solyc08g036520.2	2162	Diphosphomevalonate decarboxylase
Solyc08g036500.1	203	Orf101b
Solyc08g036440.2	159	Feruloyl transferase
Solyc08g036410.1	415	arabinogalactan protein 12
Solyc08g048160.1	504	Gamma-tubulin complex component
Solyc08g048080.1	453	Polyketide cyclase/dehydrase and lipid transport superfamily protein
Solyc08g045880.1	149	Protein YIPF
Solyc08g045870.2	177	Ribosomal protein S3
Solyc08g045860.1	155	basic helix-loop-helix (bHLH) DNA-binding superfamily protein

Solyc08g045850.3	158	pentatricopeptide repeat-containing protein
Solyc08g045750.1	161	mediator of RNA polymerase II transcription subunit
Solyc08g044610.2	164	Protein phosphatase 2C family protein
Solyc08g044440.1	144	Strubbelig receptor family protein
Solyc08g044240.1	174	Chloroplastic group IIA intron splicing facilitator CRS1, chloroplastic
Solyc08g042060.2	167	SET domain-containing protein
Solyc08g023637.1	168	Unknown protein
Solyc08g023493.1	136	Stem-specific TSJT1
Solyc08g023440.3	155	Early-responsive to dehydration stress family protein
Solyc08g023320.1	189	hAT transposon superfamily protein
Solyc08g022070.3	283	Prolyl oligopeptidase family protein
Solyc08g029260.1	1096	NADH dehydrogenase subunit 2
Solyc08g029010.1	168	Disease resistance protein (CC-NBS-LRR class) family
Solyc08g028950.2	187	DNA-directed RNA polymerase subunit beta
Solyc08g028880.2	136	30S ribosomal protein S2, chloroplastic
Solyc08g028857.1	179	Unknown protein
Solyc08g059690.1	229	AT hook motif DNA-binding family protein
Solyc08g059800.3	201	TPX2 (targeting protein for Xklp2) protein family
Solyc08g061160.2	225	BnaC09g16480D protein
Solyc08g061240.2	163	alpha/beta-Hydrolases superfamily protein
Solyc08g061493.1	149	Transposon Ty3-G Gag-Pol polyprotein
Solyc08g062275.1	149	3-oxoacyl-[acyl-carrier-protein] synthase
Solyc08g062545.1	242	Retrovirus-related Pol polyprotein from transposon TNT 1-94
Solyc08g062675.1	165	Retrovirus-related Pol polyprotein from transposon TNT 1-94
Solyc08g065163.1	167	Retrovirus-related Pol polyprotein from transposon TNT 1-94
Solyc08g067170.2	166	Retrovirus-related Pol polyprotein from transposon TNT 1-94
Solyc08g074980.3	151	Kinase family protein
Solyc08g076355.1	145	Retrovirus-related Pol polyprotein from transposon TNT 1-94
Solyc08g076550.2	272	Phosphomannomutase
Solyc08g076560.1	201	Phosphomannomutase
Solyc08g077470.3	167	Reticulon family protein
Solyc08g078800.2	210	GRAS family transcription factor
Solyc08g081750.3	177	CAAX amino terminal protease family protein
Solyc08g082120.2	145	Methanol inducible protein
Solyc08g082560.3	153	F-box SKIP8-like protein
Solyc08g082620.3	151	3-oxoacyl-[acyl-carrier-protein] synthase
Solyc09g007070.2	156	RING/U-box superfamily protein
Solyc09g008080.3	160	Ribosomal protein S5/Elongation factor G/III/V family protein
Solyc09g008750.1	153	VQ motif-containing protein, putative
Solyc09g009880.3	286	F-box family protein
Solyc09g010630.3	150	HSC2-like
Solyc09g014440.3	197	RNA polymerase II C-terminal domain phosphatase-like 2
Solyc09g014980.3	195	Protein SCAR2, putative

Solyc09g015150.1	467	Shattering 4
Solyc09g015320.2	264	Photosystem I assembly protein Ycf3
Solyc09g015440.1	138	GDT1-like protein
Solyc09g015600.2	395	Ubiquitin carboxyl-terminal hydrolase-like protein
Solyc09g015650.3	137	Non-green plastid inner envelope membrane protein
Solyc09g015880.3	170	Cytochrome c oxidase subunit 2
Solyc09g015890.3	1280	DNA-directed RNA polymerase subunit beta
Solyc09g015900.1	1133	Metallo-hydrolase/oxidoreductase superfamily protein
Solyc09g016930.1	136	Photosystem II CP43 reaction center protein
Solyc09g016957.1	277	Ubiquitin carboxyl-terminal hydrolase
Solyc09g018235.1	146	Retrovirus-related Pol polyprotein from transposon TNT 1-94
Solyc09g018630.3	154	Bis(5'-adenosyl)-triphosphatase
Solyc09g020157.1	158	MuDR family transposase containing protein
Solyc09g037180.1	154	Protein KINESIN LIGHT CHAIN-RELATED 3
Solyc09g042600.1	327	Unknown protein
Solyc09g042800.1	166	DNA-directed RNA polymerase subunit beta
Solyc09g030470.1	168	Disease resistance protein (TIR-NBS-LRR class) family
Solyc09g030453.1	159	Transposon Ty3-G Gag-Pol polyprotein
Solyc09g050020.2	851	cytochrome b
Solyc09g050030.1	490	30S ribosomal protein S14, putative
Solyc09g050040.1	671	Ribosomal protein L5
Solyc09g050050.1	364	DNA-directed RNA polymerase subunit beta
Solyc09g055210.1	144	DNA topoisomerase, type IA, core
Solyc09g055355.1	238	inhibitor/lipid-transfer protein/seed storage 2S albumin superfamily protein (DUF784)
Solyc09g055810.1	357	Protein transport protein sec23, putative
Solyc09g055840.1	192	NAD(P)H-quinone oxidoreductase subunit 2, chloroplastic
Solyc09g055950.1	249	Photosystem II D2 protein
Solyc09g056040.3	143	E3 ubiquitin-protein ligase UPL5-like protein
Solyc09g056170.3	315	Fimbrin, putative
Solyc09g056250.1	148	Orotidine 5'-phosphate decarboxylase
Solyc09g057767.1	265	ZF-HD homeobox protein
Solyc09g057810.1	219	Maturase-related protein
Solyc09g057880.3	173	Far-red impaired responsive (FAR1) family protein
Solyc09g058977.1	176	HAT family dimerisation domain containing protein
Solyc09g059045.1	1017	Retrovirus-related Pol polyprotein from transposon TNT 1-94
Solyc09g059510.3	445	Ethylene-responsive transcription factor-like protein
Solyc09g059650.3	217	Vacuolar iron transporter family protein
Solyc09g059800.1	147	Acyl-CoA N-acyltransferases (NAT) superfamily protein
Solyc09g059880.1	268	Amino acid transporter, putative
Solyc09g061390.1	137	Maturase K
Solyc09g061460.2	235	NAD(P)H-quinone oxidoreductase subunit 2, chloroplastic
Solyc09g061535.1	203	Unknown protein
Solyc09g061560.2	258	Protein OBERON 1-like protein

Solyc09g061580.1	302	Protein OBERON 1-like protein
Solyc09g061910.2	252	Calcium-dependent lipid-binding (CaLB domain) family protein
Solyc09g064430.3	163	Tyrosine decarboxylase family protein
Solyc09g064670.2	159	Leucine-rich repeat receptor-like protein kinase family protein
Solyc09g065710.1	140	Pentatricopeptide repeat-containing protein, putative
Solyc09g065770.1	140	Ubiquitin-conjugating enzyme/RWD-like protein, putative
Solyc09g065790.1	166	ATP-dependent Clp protease proteolytic subunit
Solyc09g066220.1	216	zinc ion binding/nucleic acid binding/hydrolase
Solyc09g066420.3	143	TBC1 domain family protein
Solyc09g076053.1	140	Retrotransposon protein, putative, Ty3-gypsy subclass
Solyc09g091410.1	152	Acetyl-coenzyme A carboxylase carboxyl transferase subunit beta, chloroplastic
Solyc10g005000.3	177	SUN-like protein 27
Solyc10g006910.2	171	50S ribosomal protein L16, chloroplastic
Solyc10g009070.3	278	RNA helicase DEAD32
Solyc10g012230.1	146	Protein Ycf2
Solyc10g017890.1	157	Photosystem I P700 chlorophyll a apoprotein A1
Solyc10g017920.1	156	DNA-directed RNA polymerase subunit beta
Solyc10g017950.2	156	Photosystem II reaction center protein Z
Solyc10g018198.1	179	Retrovirus-related Pol polyprotein from transposon TNT 1-94
Solyc10g018300.2	249	Transketolase
Solyc10g018520.1	146	3'-5' exonuclease domain-containing family protein
Solyc06g034317.1	169	MADS-box transcription factor family protein
Solyc10g018830.1	419	UDP-Glycosyltransferase superfamily protein
Solyc10g018840.2	208	Photosystem II CP43 reaction center protein
Solyc10g019020.1	163	Anthranilate O-methyltransferase 1
Solyc10g024415.1	196	RNA 2'-phosphotransferase, Tpt1 / KptA family
Solyc10g031550.1	386	DNA-directed RNA polymerase subunit beta
Solyc10g031560.1	273	DNA-directed RNA polymerase subunit
Solyc10g036620.2	156	UDP-D-glucose/UDP-D-galactose 4-epimerase 3
Solyc10g037990.1	160	Maturase K
Solyc10g039290.2	151	Nitrate transporter protein 1.2-like protein
Solyc10g061830.2	155	Photosystem II CP43 reaction center protein
Solyc10g061980.1	360	Neutral/alkaline invertase
Solyc10g061990.2	498	NADH-ubiquinone oxidoreductase chain 4
Solyc10g062117.1	156	Retrovirus-related Pol polyprotein from transposon TNT 1-94
Solyc10g044540.2	143	ATP synthase subunit alpha, chloroplastic
Solyc10g044547.1	602	Formin-like protein
Solyc10g044900.2	184	Ion channel DMI1
Solyc10g044993.1	165	Retrovirus-related Pol polyprotein from transposon TNT 1-94
Solyc10g045190.2	211	RNA-DIRECTED DNA METHYLATION 1
Solyc10g045290.2	218	Kinase interacting (KIP1-like) family protein, putative
Solyc10g045350.2	139	ecotropic viral integration site protein
Solyc10g045690.1	143	Gibberellin 20-oxidase

Solyc10g045750.1	212	NADH-ubiquinone oxidoreductase chain 4
Solyc10g046770.2	148	Trehalose-6-phosphate synthase, putative
Solyc10g046777.1	139	Retrovirus-related Pol polyprotein from transposon 297
Solyc10g046840.1	179	Starch synthase, chloroplastic/amyloplastic
Solyc10g046990.2	331	Ribulose bisphosphate carboxylase small chain, chloroplastic
Solyc10g047030.3	164	LEXYL1 protein
Solyc10g047410.1	214	Photosystem II CP43 reaction center protein
Solyc10g047490.1	162	Leucine-rich repeat receptor-like protein kinase family protein
Solyc10g048060.1	168	myosin XI D
Solyc10g048065.1	1668	Transport membrane protein
Solyc10g049300.2	160	Glycine-rich protein A3
Solyc10g049360.2	147	cyclin A3_6
Solyc10g049470.1	157	Ycf1
Solyc10g049590.1	255	DNA-directed RNA polymerase subunit beta
Solyc10g049940.1	222	Myb/SANT-like DNA-binding domain protein
Solyc10g052740.2	142	Photosystem I P700 chlorophyll a apoprotein A1
Solyc10g052770.1	269	DNA/RNA polymerases superfamily protein
Solyc10g052775.1	168	Retrovirus-related Pol polyprotein from transposon 297 family
Solyc10g055020.2	146	Mediator of RNA polymerase II transcription subunit 20-like protein
Solyc10g076430.1	152	Pectinesterase
Solyc10g077131.1	136	Invertase/pectin methylesterase inhibitor family protein
Solyc10g079750.2	144	BTB/POZ ankyrin repeat protein
Solyc10g080393.1	168	2-oxoglutarate (2OG) and Fe(II)-dependent oxygenase superfamily protein
Solyc11g012930.2	254	WAT1-related protein
Solyc11g013130.2	154	cysteine-rich/transmembrane domain protein A
Solyc11g013740.2	152	Guanylate-binding family protein
Solyc11g013790.2	148	Tim10/DDP family zinc finger protein
Solyc11g018700.2	172	Ycf15 protein
Solyc11g020347.1	185	Retrovirus-related Pol polyprotein from transposon TNT 1-94
Solyc11g020353.1	141	Adenylate isopentenyltransferase
Solyc11g020490.2	262	40S ribosomal protein S27
Solyc11g020785.1	159	Retrovirus-related Pol polyprotein from transposon TNT 1-94
Solyc11g021100.1	450	NAD(P)H-quinone oxidoreductase subunit 2, chloroplastic
Solyc11g021120.1	448	NAD(P)H-quinone oxidoreductase subunit 2, chloroplastic
Solyc11g021160.1	241	Ycf68
Solyc11g021180.1	185	Ycf1
Solyc11g021210.1	152	Cytochrome c biogenesis protein CcsA
Solyc11g021240.2	149	Ycf1
Solyc11g021270.1	169	Ycf1
Solyc11g021290.2	140	Ycf1
Solyc11g021300.1	154	Ycf1
Solyc11g022410.1	727	Homeobox leucine zipper protein
Solyc11g022530.2	166	Late embryogenesis abundant protein (LEA) family protein

Solyc11g022610.2	148	30S ribosomal protein S4, chloroplastic
Solyc11g050900.2	221	Sulfotransferase
Solyc11g051005.1	178	Pectinesterase
Solyc11g051035.1	338	pre-mRNA-splicing factor of RES complex protein
Solyc11g051170.2	195	ATP synthase subunit alpha, chloroplastic
Solyc11g051200.1	165	Senescence-associated protein
Solyc11g027630.1	164	glycosyl hydrolase 9A3
Solyc11g027640.1	160	phosphatidylinositol 4-OH kinase beta1
Solyc11g027645.1	171	Ribosomal RNA small subunit methyltransferase B
Solyc11g027665.1	186	Retrovirus-related Pol polyprotein from transposon 17.6
Solyc11g027690.1	192	Flavin-binding kelch domain F box protein
Solyc11g027693.1	144	Retrovirus-related Pol polyprotein from transposon 17.6
Solyc11g027697.1	160	Retrovirus-related Pol polyprotein from transposon 17.6
Solyc11g027760.1	164	Cytochrome P450 like_TBP
Solyc11g027770.1	172	Senescence-associated protein
Solyc11g028123.1	473	transmembrane protein
Solyc11g028127.1	297	Retrovirus-related Pol polyprotein from transposon TNT 1-94
Solyc11g028150.1	183	Retrovirus-related Pol polyprotein from transposon TNT 1-94
Solyc11g028160.1	173	Cytochrome c biogenesis B
Solyc11g030570.1	339	NADH-ubiquinone oxidoreductase chain 4
Solyc11g030810.1	159	Polynucleotidyl transferase, ribonuclease H-like superfamily protein
Solyc11g030900.1	280	Maturase-related protein
Solyc11g030903.1	847	NADH-ubiquinone oxidoreductase chain 5
Solyc11g045450.2	361	B3 domain-containing protein family
Solyc11g045440.1	297	delta(3), delta(2)-enoyl CoA isomerase 1
Solyc11g045430.1	226	transmembrane protein
Solyc11g045150.1	205	myosin XI D
Solyc11g044630.1	144	Calcium-dependent protein kinase
Solyc11g042650.1	251	Target of Myb protein 1
Solyc11g039980.2	578	ATP synthase subunit alpha
Solyc11g039970.1	752	Retrovirus-related Pol polyprotein from transposon TNT 1-94
Solyc11g039910.2	144	Mitochondrial import inner membrane translocase subunit Tim17-like protein
Solyc11g039860.2	150	Tetratricopeptide repeat (TPR)-like superfamily protein
Solyc11g039730.1	233	NAD(P)H-quinone oxidoreductase subunit K, chloroplastic
Solyc11g039660.1	150	Actin
Solyc11g039640.2	203	chromatin remodeling factor CHD3 (PICKLE)
Solyc11g039390.1	1013	calpain-type cysteine protease family
Solyc11g039380.1	1553	TRICHOME BIREFRINGENCE-LIKE 9
Solyc11g039370.2	1584	Glycosyl hydrolase superfamily protein
Solyc11g039360.1	445	Cytochrome c biogenesis FC
Solyc11g039350.1	277	mitochondrial editing factor 19
Solyc11g038350.1	1281	Pentatricopeptide repeat (PPR) superfamily protein
Solyc11g038340.1	3163	Nuclear control of ATP synthase 2

Solyc11g056250.1	138	glutamine-dependent asparagine synthase 1
Solyc11g056270.2	267	Maturase
Solyc11g056280.1	384	1,2-alpha-L-fucosidase
Solyc11g056290.2	1274	DNA-directed RNA polymerase
Solyc11g056310.1	1132	Ribosomal protein S13
Solyc11g056330.1	818	cysteine-rich RLK (RECEPTOR-like protein kinase) 19
Solyc11g056340.1	799	Photosystem II protein D1
Solyc11g056350.1	1499	RHOMBOID-like protein 4
Solyc11g056360.2	1043	Ribosomal protein S12
Solyc11g056380.1	1396	Protein transport protein sec23, putative
Solyc11g056390.1	517	30S ribosomal protein S7, chloroplastic
Solyc11g056400.2	720	Ribosomal protein S3
Solyc11g056410.2	277	Cytochrome c oxidase subunit 2
Solyc11g056440.1	1517	ureide permease 2
Solyc11g056460.1	480	Retrovirus-related Pol polyprotein from transposon TNT 1-94
Solyc11g056470.2	655	RING/U-box superfamily protein
Solyc11g056500.2	436	OR338
Solyc11g056510.1	998	Ribonuclease H
Solyc11g056530.1	443	RNA-directed DNA polymerase (Reverse transcriptase)
Solyc11g061980.1	1402	Glycosyltransferase
Solyc11g062070.2	153	Outer envelope protein 80, chloroplastic
Solyc11g062350.2	295	Blue copper protein
Solyc11g063480.1	294	delta(3), delta(2)-enoyl CoA isomerase 1
Solyc11g063490.1	155	Glucose-6-phosphate isomerase, cytosolic 1
Solyc11g063500.1	447	nitrite reductase 1
Solyc11g063520.1	573	sequence-specific DNA binding transcription factor ATNDX
Solyc11g063540.1	369	Photosystem II CP43 reaction center protein
Solyc11g063570.1	143	F-box/RNI-like superfamily protein
Solyc11g063580.2	274	Photosystem II stability/assembly factor HCF136, chloroplastic
Solyc11g063600.2	628	Cytochrome c oxidase subunit 1
Solyc11g063620.2	262	Cytochrome c biogenesis FN
Solyc11g063640.1	662	Maturase K
Solyc11g063650.1	183	Adenylate isopentenyltransferase
Solyc11g063740.2	162	Adenylate isopentenyltransferase
Solyc11g064940.1	159	AMP-dependent synthetase and ligase family protein
Solyc11g065360.2	275	ABC transporter G family member 15
Solyc11g069020.2	157	Disease resistance protein
Solyc12g006350.2	344	Auxin response factor
Solyc12g006860.2	187	brassinosteroid hydroxylase
Solyc12g008890.2	181	Cytokinin oxidase/dehydrogenase-like
Solyc12g011460.1	527	Cyclophilin-like peptidyl-prolyl cis-trans isomerase family protein
Solyc12g014320.2	216	Anaphase-promoting complex subunit 6-like protein
Solyc12g014600.2	238	RNA-binding protein, putative
Solyc12g016125.1	267	Protein kinase

Solyc12g017700.2	138	ATP-dependent RNA helicase
Solyc12g017750.2	160	pentatricopeptide (PPR) repeat-containing protein
Solyc12g017885.1	466	DNA-directed RNA polymerases II and IV subunit 5A
Solyc12g019627.1	150	Cytochrome P450
Solyc12g019940.1	148	Polyprotein
Solyc12g021360.2	743	Protein apaG
Solyc12g082700.1	188	ABC(ABCB) family transporter: mitochondrial ATM1-like protein (ABCB)
Solyc12g082790.1	137	Bifunctional dihydroflavonol 4-reductase/flavanone 4-reductase
Solyc12g077540.2	138	Trihelix transcription factor GT-1
Solyc12g077546.1	143	Retrovirus-related Pol polyprotein from transposon TNT 1-94
Solyc12g026400.2	136	Protease Do-like 9
Solyc12g027550.1	196	Photosystem II CP43 reaction center protein
Solyc12g027753.1	265	Ribosomal protein S12
Solyc12g027765.1	152	Retrotransposon protein, putative, Ty3-gypsy subclass
Solyc12g027815.1	144	Retrovirus-related Pol polyprotein from transposon 17.6
Solyc12g027820.1	191	NAD(P)H-quinone oxidoreductase subunit 2, chloroplastic
Solyc12g070100.2	146	Mediator of RNA polymerase II transcription subunit 25
Solyc12g070220.2	181	Protein Ycf2
Solyc12g070270.2	149	ABC transporter B family protein
Solyc12g062450.2	154	DNA repair metallo-beta-lactamase family protein
Solyc12g062470.2	386	Protein Ycf2
Solyc12g062480.2	191	conserved telomere maintenance component 1
Solyc12g062530.2	167	ATP synthase beta subunit
Solyc12g062810.1	179	glucose-6-phosphate/phosphate translocator 2
Solyc12g063010.2	225	ARID/BRIGHT DNA-binding domain-containing protein
Solyc12g032990.2	301	Photosystem I P700 chlorophyll a apoprotein A2
Solyc12g033030.1	185	50S ribosomal protein L23, chloroplastic
Solyc12g033090.2	201	50S ribosomal protein L23, chloroplastic
Solyc12g035475.1	203	Endoplasmic reticulum metallopeptidase 1
Solyc12g035524.1	155	Retrovirus-related Pol polyprotein from transposon TNT 1-94
Solyc12g035550.1	330	Ycf1
Solyc12g035620.2	354	Holliday junction resolvase-like protein
Solyc12g035670.2	139	SAC3/GANP/Nin1/mts3/eIF-3 p25 family
Solyc12g035826.1	581	Cytochrome c biogenesis C
Solyc12g035828.1	164	50S ribosomal protein L23, chloroplastic
Solyc12g035860.1	163	30S ribosomal protein S11, chloroplastic
Solyc12g035865.1	263	ARF GAP-like zinc finger-containing protein ZIGA4
Solyc12g035870.2	139	DNA-directed RNA polymerase subunit beta
Solyc12g035875.1	157	30S ribosomal protein S3, chloroplastic
Solyc12g035920.1	166	NADH-ubiquinone oxidoreductase chain 6
Solyc12g035930.1	153	DNA-directed RNA polymerase subunit beta
Solyc12g036350.1	157	nudix hydrolase homolog 10
Solyc12g036415.1	156	SAUR-like auxin-responsive protein family

Solyc12g036550.2	138	Ycf1
Solyc12g036640.1	146	Extracellular calcium sensing receptor
Solyc12g036677.1	164	Cytochrome P450
Solyc12g036710.2	195	RNA polymerase subunit
Solyc12g036720.1	143	Maturase
Solyc12g036870.2	224	Myb/SANT-like DNA-binding domain protein
Solyc12g037940.2	194	DNA-directed RNA polymerase subunit beta
Solyc12g038000.2	179	Peptidyl-prolyl cis-trans isomerase
Solyc12g038657.1	138	Protein FAR1-RELATED SEQUENCE 5
Solyc12g042230.2	283	ATP synthase subunit beta, chloroplastic
Solyc12g044440.2	171	Zinc finger protein
Solyc12g044850.2	352	WAS/WASL-interacting family protein
Solyc12g088410.2	378	Glycosyl hydrolase family 35 protein, putative
Solyc12g088540.2	252	WD40 repeat-like protein
Solyc12g098100.2	201	Non-specific serine/threonine protein kinase
